# The anoxic electrode‐driven fructose catabolism of *Pseudomonas putida* KT2440

**DOI:** 10.1111/1751-7915.13862

**Published:** 2021-06-11

**Authors:** Anh Vu Nguyen, Bin Lai, Lorenz Adrian, Jens O. Krömer

**Affiliations:** ^1^ Department of Solar Materials Helmholtz Centre for Environmental Research ‐ UFZ Leipzig Germany; ^2^ Department of Environmental Biotechnology Helmholtz Centre for Environmental Research ‐ UFZ Leipzig Germany; ^3^ Chair of Geobiotechnology Technische Universität Berlin Berlin Germany

## Abstract

*Pseudomonas putida* (*P. putida*) is a microorganism of interest for various industrial processes, yet its strictly aerobic nature limits application. Despite previous attempts to adapt *P. putida* to anoxic conditions via genetic engineering or the use of a bioelectrochemical system (BES), the problem of energy shortage and internal redox imbalance persists. In this work, we aimed to provide the cytoplasmic metabolism with different monosaccharides, other than glucose, and explored the physiological response in *P. putida* KT2440 during bioelectrochemical cultivation. The periplasmic oxidation cascade was found to be able to oxidize a wide range of aldoses to their corresponding (keto‐)aldonates. Unexpectedly, isomerization of the ketose fructose to mannose also enabled oxidation by glucose dehydrogenase, a new pathway uncovered for fructose metabolism in *P. putida* KT2440 in BES. Besides the isomerization, the remainder of fructose was imported into the cytoplasm and metabolized. This resulted in a higher NADPH/NADP^+^ ratio, compared to glucose. Comparative proteomics further revealed the upregulation of proteins in the lower central carbon metabolism during the experiment. These findings highlight that the choice of a substrate in BES can target cytosolic and periplasmic oxidation pathways, and that electrode‐driven redox balancing can drive these pathways in *P*. *putida* under anaerobic conditions.

## Graphical abstract

A new pathway was uncovered for the fructose metabolism in *Pseudomonas putida* KT2440 cultivated anaerobically in a bioelectrochemical system. In addition to the typical PTS transporter‐based pathway observed under aerobic condition, fructose was firstly converted to mannose by isomerization and then oxidized to mannonate by the glucose dehydrogenase.

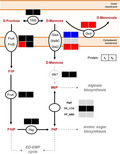

## Introduction

*Pseudomonas putida* has previously demonstrated hallmark features of an interesting microbial chassis for industrial and environmental processes (Wackett, 2003; Poblete‐Castro *et al*., 2012; Nikel *et al*., 2014; Loeschcke and Thies, 2015), due to its versatile metabolism towards a wide range of carbon sources (La Rosa *et al*., 2016; Molina *et al*., 2019) and high resistance to environmental stresses (Nikel *et al*., 2016). Its central carbon metabolism includes three different glycolytic pathways, that is the Embden‐Meyerhof‐Parnas (EMP) pathway with the exception of 6‐phosphofructose kinase, the Entner–Doudoroff (ED) pathway and the oxidative pentose phosphate (PP) pathway (Fig. S1) (Chavarría *et al*., 2013; Nikel *et al*., 2016). All three constitute a cyclic pathway, which was recently termed the ED–EMP cycle (Nikel *et al*., 2015; Kohlstedt and Wittmann, 2019). The ED–EMP cycle is an important pathway to regenerate NADPH while *P*. *putida* suffers from oxidative stress (Olavarria *et al*., 2015; Nikel et al., 2020), and overall leads to the central metabolite glyceraldehyde 3‐phosphate.

In larger scale bioproduction or remediation, aeration can become cost‐intensive (Stephanopoulos *et al*., 1998; Huang and Tang, 2007; Ghangrekar and Behera, 2014) and facultative anaerobes, such as *Escherichia coli*, might form large amounts of by‐products, such as acetate, which can inhibit growth and fermentation performance of the bacteria (Takahashi *et al*., 1999; De Mey *et al*., 2007; Pinhal *et al*., 2019). As an obligate aerobe, *P. putida* does not form such fermentative by‐products, but *P. putida* requires oxygen not only as an electron sink but also as a reagent of oxygenation reactions. Recently, oxygen‐dependent enzymes essential for biomass production were replaced with non‐oxygen‐associated alternatives (Kampers *et al*., 2019). Nevertheless, the published efforts to include selected fermentative or anaerobic respiratory pathways to establish anaerobic electron balancing in the cells (Sohn *et al*., 2010; Nikel and de Lorenzo, 2013; Steen *et al*., 2013) still relied on fermentation by‐products or co‐substrates as electron sinks. Although these strategies were able to improve the bacterium’s survival under anoxic conditions, no substantial anaerobic growth was observed, along with a persisting problem of energy shortage and redox imbalance.

An approach to decouple the electron balance from the carbon balance, or depletable external electron acceptors is the use of microbial electrochemical technologies (METs). Here, electrodes take up excess electrons from respiratory pathways or fermentation reactions. In recent years, various bioelectrochemical system (BES) designs have been utilized for anodic cultivation of *P. putida* under oxygen‐deprived conditions. In brief, an electrode (anode) was provided as the terminal electron sink for the cells via redox mediators, such as ferricyanide Fe[CN]_6_^3‐^ (Hintermayer *et al*., 2016; Lai *et al*., 2016b; Yu *et al*., 2018), or self‐secreted phenazines (Schmitz *et al*., 2015; Askitosari *et al*., 2019) to drive anaerobic carbon metabolism. The recorded current response provides a direct measurement of catabolic reactions occurring in the system, as electrons are released during substrate oxidation.

In the case of *P. putida* strains F1 and KT2440 cultivated in a BES, no growth was detected when glucose was used as substrate; the carbon flux was dominated by the periplasmic enzymes glucose dehydrogenase (Gcd) and gluconate dehydrogenase complex (Gad) (Yu *et al*., 2018), which are together referred to as the periplasmic oxidation cascade (POC), while the cytoplasmic metabolic activities were minor. The cells also showed a significant decrease of the NADPH/NADP^+^ ratio over the cultivation time (Lai *et al*., 2016b), which might have been simultaneously a consequence of carbon flux redirection away from the ED–EMP cycle, and/or a cause of the limited carbon uptake under BES condition. Such unbalanced redox metabolism and limited cytosolic carbon uptake could possibly be the main constraint limiting the anaerobic metabolism (or even the anaerobic growth) of *P. putida* cells in BES. Revealing the reason behind this will be of great significance for BES process development.

In this study, we aimed to tackle the redox metabolism and carbon uptake limitations by exploiting different carbohydrates, other than glucose, for *P. putida* KT2440 in BES. Glucose was mainly taken up via the POC pathway while PQQ and FAD are involved as the main redox cofactors (Nikel *et al*., 2015), other carbohydrates catabolized via different pathways may force the cells into a different redox status. We firstly tested several aldoses and found that all of them were oxidized via POC, suggesting a broad specificity of the cascade towards aldoses. Therefore, fructose, a ketose that was aerobically taken up by *P. putida* via a phosphotransferase system and oxidized directly in the cytosol (Chavarría *et al*., 2012), was then used for further studies. Surprisingly, we found fructose was also partly metabolized through POC while fructose was isomerized to mannose and then oxidized to mannonate. The remaining fructose was metabolized via the cytosolic pathway and improved the redox status (particular NADPH/NADP^+^ ratio) of *P. putida* cells in BES. This study demonstrates how versatile the possible oxidation processes in a BES with *P*. *putida* are and that the electrodes can influence the intracellular redox balance under various conditions.

## Results

### Aldoses are oxidized in the periplasm to the corresponding aldonic acids

Several monosaccharides were tested to identify which sugars can be taken up by KT2440 under BES conditions. In addition to d‐glucose, d‐fructose and d‐ribose can be used for *P. putida* KT2440’s aerobic cultivation, although a slower growth was observed for both substances (Fig. S2A). On the other hand, d‐galactose and l‐arabinose did not support the growth of KT2440 aerobically, yet these two sugars were found to be oxidized to their corresponding aldonates under aerobic conditions (Fig. S2B–E).

**Fig. 1:**
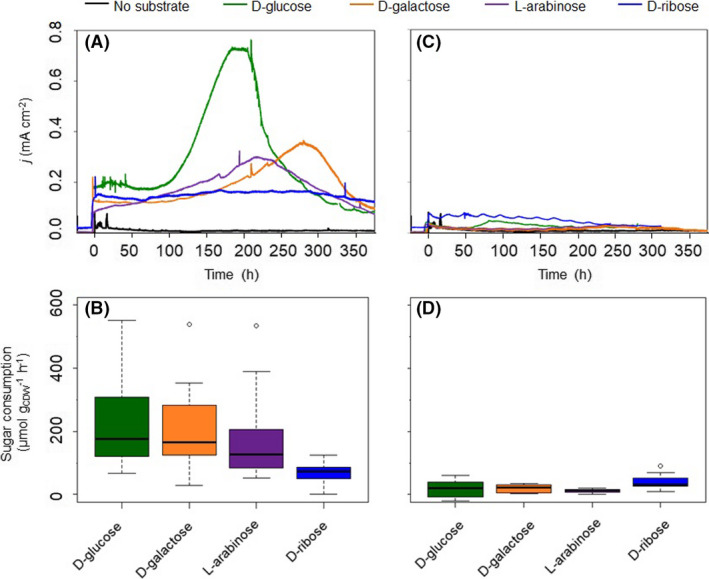
Comparative performance of KT2440 and KT2440*Δgcd* in BES with different aldose substrates. Current densities of (A) KT2440 and (C) KT2440*Δgcd* cultures in BES with various aldoses (*n* = 1) versus no‐substrate control (*n* = 1), and box plots of the sugar consumption rate per g_CDW_ per h of (B) KT2440 and (D) KT2440*Δgcd* between at least six sampling points during BES cultivation of each sugar (*n* ≥ 5). For each box plot, whiskers indicate values outside the upper and lower quartiles; outliers are plotted as individual points.

When cultivated in a BES in which the anode provided a surface to deposit electrons and where the electrode potential and current between the electrodes were continuously monitored, *P. putida* KT2440 cultures fed with d‐glucose produced the highest peak current, followed by cultures with d‐galactose, l‐arabinose or d‐ribose (Fig. 1A), yet no cell growth was detected in any of the cultures (Fig. S3A). Concurrent with the current generation, the concentrations of the sugars continuously decreased over time (Fig. 1B, Fig. S3B). The sugars’ corresponding aldonates and 2‐ketoaldonates were subsequently identified by GC‐MS as the main end‐products (Fig. S4). Compared to the wild type strain, the glucose dehydrogenase knockout mutant of KT2440 (KT2440Δ*gcd*) showed significantly lower current outputs in BES when either d‐glucose, d‐galactose or l‐arabinose was fed. Sugar consumption rates were also significantly decreased (Fig. 1C and D, Fig. S3C and D). This indicates that the carbohydrates were primarily metabolized through the POC of *P. putida* KT2440 and that the electrons from sugar oxidation were transferred to the anode. HPLC analyses of the respective samples also showed that in KT2440Δ*gcd* the UV absorption spectra corresponding to aldonates and 2‐ketoaldonates were reduced or absent, compared to in KT2440 (data not shown), further validating the function of POC pathway in consuming the aldoses.

### Fructose is partially isomerized to mannose in the periplasm

As demonstrated above, aldoses were mainly oxidized via the POC, and thus, may not provide sufficient cytoplasmic substrates to drive energy and redox metabolism. Therefore, fructose was subsequently tested as a substrate since (i) the sugar is a ketose, and (ii) KT2440 has a specialized phosphotransferase system (PTS) for its uptake (Sawyer *et al*., 1977). The PTS system is not NAD(P)H‐dependent, a metabolic constraint observed previously for *P. putida* F1 on glucose under BES conditions (Lai *et al*., 2016b). Here, we assessed the bioelectrochemical performance of KT2440 alongside analysis of the metabolites in the medium, then compared it with strain KT2440Δ*gcd*. A control experiment without inoculum showed that fructose concentration in the medium did not significantly change after more than 500 h (data not shown).

**Fig. 2:**
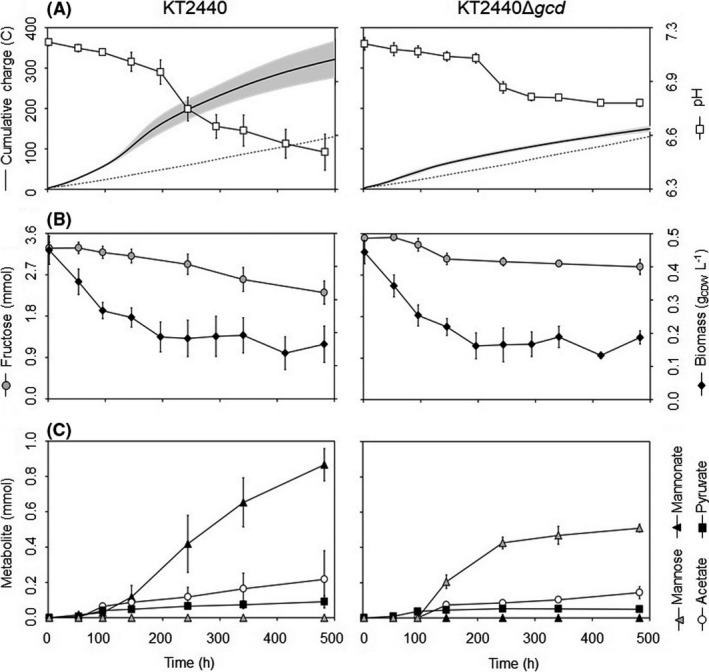
Comparative performance of KT2440 and KT2440*Δgcd* in BES with fructose as substrate. (A) Cumulative charge (solid line) versus no‐cell control (*n* = 1, dot line) and medium pH (white square); (B) Biomass (black diamond) and total fructose level (grey circle); (C) Mannose (gray triangle), mannonate (black triangle), acetate (white circle), and pyruvate (black square) level of KT2440 (left column, *n* = 3) and KT2440Δ*gcd* (right column, *n* = 3) cultures observed during BES cultivation. Data are averages of biological triplicates; grey areas/error bars represents the standard deviation of sample.

Both the wild‐type KT2440 and the KT2440Δ*gcd* mutant showed a similar decreasing trend in biomass density in BES and there was no significant difference between them over the cultivation period (Student’ t‐test yielded p‐values > 0.05 at all sampling points). The fructose consumption was 0.97 ± 0.13 and 0.62 ± 0.11 mmol for the respective strain over this period (Fig. 2B). Interestingly, KT2440 produced in total 1.98 ± 0.46 mmol of electrons in comparison with only 0.34 ± 0.04 mmol of electrons for KT2440*Δgcd* (Fig. 2A). At the same time, the pH drop in the medium was also steeper in strain KT2440 compared to KT2440Δ*gcd*. The Gcd, and thus POC, was involved in fructose metabolism, although the current output in *P. putida* KT2440Δ*gcd* mutant indicate a second metabolic pathway existing as well, as more electrons were produced during the first 244 h compared to the background current. In order to clarify the nature of POC for fructose metabolism, the extracellular metabolites of the KT2440 and KT2440Δ*gcd* cultures were analysed with GC‐MS. In the KT2440 cultures, mannonate – a stereoisomer at the C‐2 position of gluconate – was found as the sole extracellular aldonate, while in the KT2440Δ*gcd* cultures, mannose was detected instead of mannonate (Fig. S5). Strain KT2440 produced 0.87 ± 0.09 mmol mannonate, and KT2440Δ*gcd* produced 0.51 ± 0.02 mmol of mannose. A constant mannose level in KT2440Δ*gcd* cultures after 244 h was observed, whereas mannonate continued to build up in KT2440 cultures (Fig. 2C).

In addition to the POC discussed above, a second metabolic pathway might be present to catabolize fructose in BES, indicated by both the current output and the pH drop for the *P. putida* KT2440Δ*gcd*. The carbon balance was closed under the assumption that per mol acetate formed one mol of CO_2_ was produced (Table 1). This is an acceptable assumption for hexose metabolism to acetate in *P. putida*, since decarboxylation is expected (Lai *et al*., 2016b). Since planktonic biomass decreased over time (Fig. 2B) and no significant amount of biomass formation was observed on the electrode surface (Fig. S6), biomass was omitted from the calculation. Product yields and specific rates were calculated using the average planktonic biomass concentration throughout the processes. Interestingly, strain KT2440 consumed fructose twice as fast compared to its Δ*gcd* counterpart (Table 1). Y_mannose_ and r_mannose_ were undetermined for strain KT2440 as mannose was not detectable in these cultures. Regarding the electron fluxes, 87.6% of the measured electrons were associated with the POC activity for KT2440 (with an estimation of 1.7 ± 0.2 mmol of electron released from fructose oxidation to mannonate, in comparison with 2.0 ± 0.5 mmol of captured electrons). On the other hand, the amounts of electrons produced by KT2440Δ*gcd* by an unidentified, but likely to be cytoplasmic, pathway were 0.34 ± 0.04 mmol during the first 244 h, since the isomerization between fructose and mannose would not generate electrons.

**Table 1:** Process parameters of anoxic fructose conversion of KT2440 and KT2440Δ*gcd* in BES reactors.

	KT2440	KT2440Δ*gcd*
Carbon balance (%)	101.9 ± 1.7	102.4 ± 3.3
Coulombic efficiency (%)	85.0 ± 5.4	93.5 ± 18.0^a^
Yield (mol_product_ mol_fructose_^‐1^)		
Y_mannose_	ND	0.820 ± 0.130
Y_mannonate_	0.934 ± 0.127	0
Y_acetate_	0.192 ± 0.102	0.204 ± 0.035
Y_pyruvate_	0.079 ± 0.018	0.073 ± 0.028
Rate (µmol g_CDW_^‐1^ h^‐1^)		
r_fructose_	−24.05 ± 4.06	−11.95 ± 3.18
r_mannose_	ND	9.69 ± 2.27
r_mannonate_	22.77 ± 6.63	0
r_acetate_	4.34 ± 1.48	2.46 ± 0.84
r_pyruvate_	1.86 ± 0.29	0.86 ± 0.34

ND, not determined.

Data are average from biological triplicates (*n* = 3); the values are depicted as “mean ± standard deviation of sample.

^a^ Value determined at 244 h after inoculation.

### Fructose uptake affects the internal energy and redox state of KT2440

In order to investigate how fructose uptake and utilization affected the energy and redox status of the cell under BES condition, the ATP/ADP and the NAD(P)H/NA(P)D^+^ ratios were measured using protocol similar to Lai *et al*. (2016b). ATP/ADP ratio of KT2440 in BES was 3.6‐times lower compared to *P. putida* F1 in BES (Table 2). Furthermore, the ATP/ADP ratio of KT2440 was also more than twice that of strain KT2440Δ*gcd*. Regarding the redox ratios, when KT2440 was cultivated in BES with fructose, NADPH/NADP^+^ increased throughout the cultivation, and the NADPH/NADP^+^ value at around 170 h after inoculation (which was approximately 24 h after peak current was reached) was around 8‐fold higher compared to that of strain F1 cultivated on glucose at peak current (Table 2). In contrast, the NAD pool was found to shift more toward the oxidized form when the cells were fed with fructose in BES, differing from a relatively stable NADH/NAD^+^ ratio in the case of glucose amongst anoxic settings (Table 2, Fig. S7).

**Table 2:** Comparison of energy and redox ratios of *P. putida* cultivated in aerobic and anoxic conditions.

Strain	Substrate	Condition	ATP/ADP (mol mol^‐1^)	NADH/NAD^+^ (mol mol^‐1^)	NADPH/NADP^+^ (mol mol^‐1^)	References
F1	Glucose	Anaerobic^a^ BES, peak current	2.37–4.18 3.73	0.20–0.22 0.22	0.09–0.18 0.08	Lai *et al*. (2016b) Lai *et al*. (2016b)
KT2440	Fructose	BES, peak current	1.04 ± 0.53	0.07 ± 0.01	0.49 ± 0.06	This study
KT2440Δ*gcd*	Fructose	BES, peak current	0.50 ± 0.13	ND.	ND.	This study

ND, not determined.

Data are average from biological triplicates (*n* = 3); the values are depicted as “mean ± standard deviation of sample.

^a^including cultures in anaerobic bottles and open‐circuit BES setups.

### BES cultivation of KT2440 in fructose enables synthesis of the central carbon metabolism’s proteins

To explore the effect of anaerobic metabolism of fructose on the central metabolism, protein expression of strain KT2440 during BES cultivation was investigated. In total, 2377 different proteins were detected across all samples, covering 43.5% of the predicted open reading frames in KT2440 (Nelson *et al*., 2002; Belda *et al*., 2016). The threshold for expression fold change and p‐value were 2 and 0.05, respectively, that is expressions increasing or decreasing by more than two times, in comparison with the reference point t_0_, were considered significant. Normalized data from 146 h (peak current, t_1_) and 414 h (t_2_) after inoculation (Fig. S8A) showed that 479 and 212 proteins were significantly upregulated, and 56 and 95 proteins were significantly downregulated, respectively, compared to cultures at the time of inoculation (t_0_) (Fig. S8B and C). Proteins that could not be quantified at all time points were excluded from the volcano plots as their normalized‐to‐t_0_ log fold change could not be calculated. This affected on the one hand 200 and 393 proteins at t_1_ and t_2_, respectively, that could not be quantified for t_0_ and on the other hand 348 and 276 t_0_ proteins, which could not be quantified at t_1_ and t_2_, respectively. Similarly, an open‐circuit experiment (i.e., no potential applied on the anode) with two biological replicates was conducted as control, resulting in 1753 different proteins detected across all samples; only 7 and 15 proteins were upregulated, and 90 and 62 proteins were downregulated at the respective sampling points of the BES experiment (Fig. S8D and E).

**Fig. 3:**
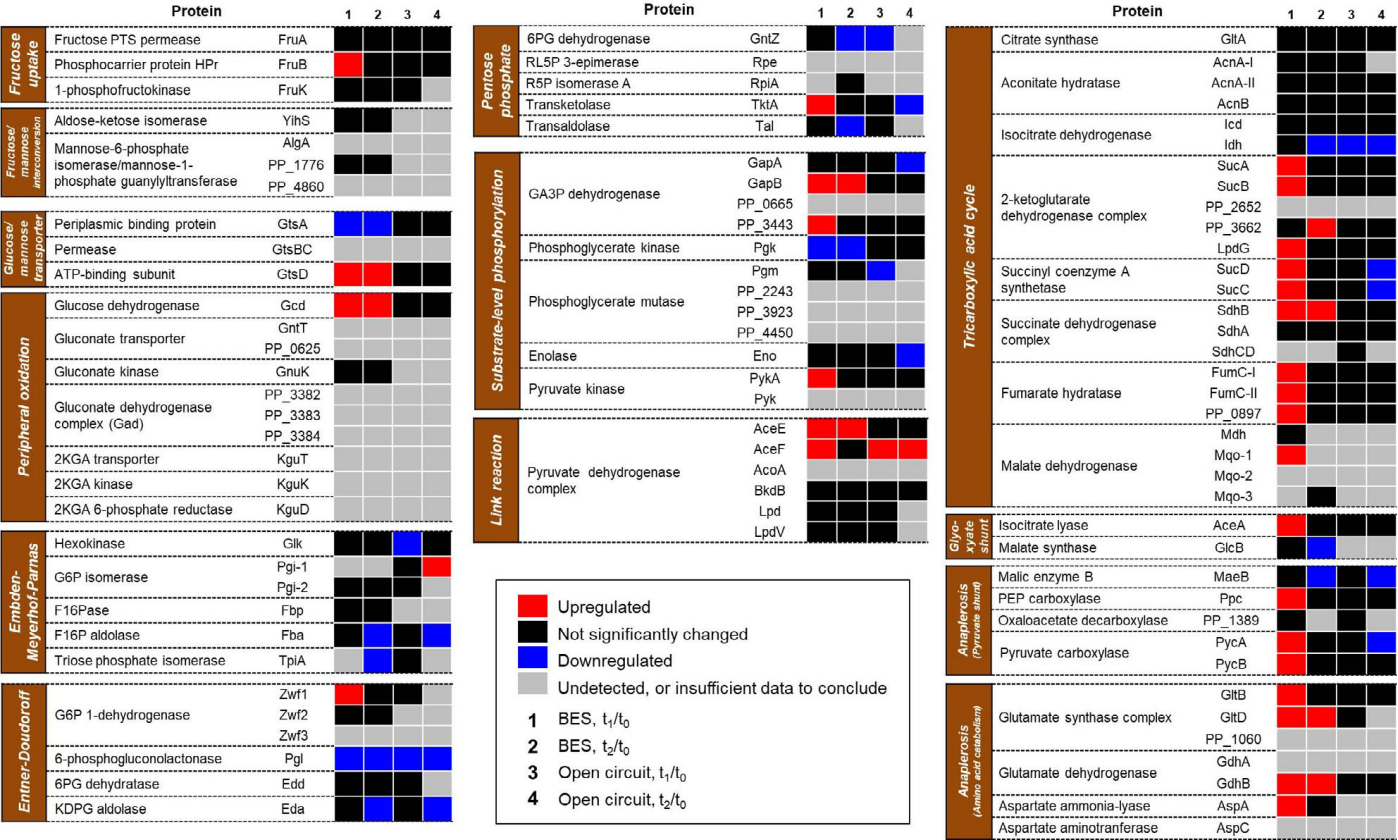
Proteome analysis of KT2440’s central carbon metabolism during BES cultivation in fructose. Differential expression analysis of the proteins involved in the central carbon metabolism pathway during KT2440’s BES (*n* = 3) and open‐circuit (*n* = 2) cultivation in fructose. 1, change of expression at peak current (t_1_) compared to at inoculation (t_0_) during BES cultivation; 2, change of expression 414 h after inoculation (t_2_) compared to at inoculation during BES cultivation; 3, change of expression at 146 h (t_1_) compared to at inoculation (t_0_) during open‐circuit cultivation; 4, change of expression 414 h after inoculation (t_2_) compared to at inoculation during open‐circuit cultivation. Abundance of individual protein is normalized to abundance of GapDH Mrsa252. Statistical analysis of protein abundance over the course of the experiment was conducted using one‐tailed paired t‐test. Data with ∣log_2_FC∣ > 1 and *P*‐value < 0.05 are considered to be significant. Red indicates upregulation, blue indicates downregulation, black indicates insignificant change, grey indicates FC not determined. 2KGA, 2‐ketogluconate; G6P, glucose 6‐phosphate; F16P, fructose 1,6‐bisphosphate; 6PG, 6‐phosphogluconate; KDPG, 2‐keto‐3‐deoxy‐6‐phosphogluconate; RL5P, ribulose 5‐phosphate; R5P, ribose 5‐phosphate; GA3P, glyceraldehyde 3‐phosphate; PEP, phosphoenolpyruvate; Aa, amino acid.

**Fig. 4:**
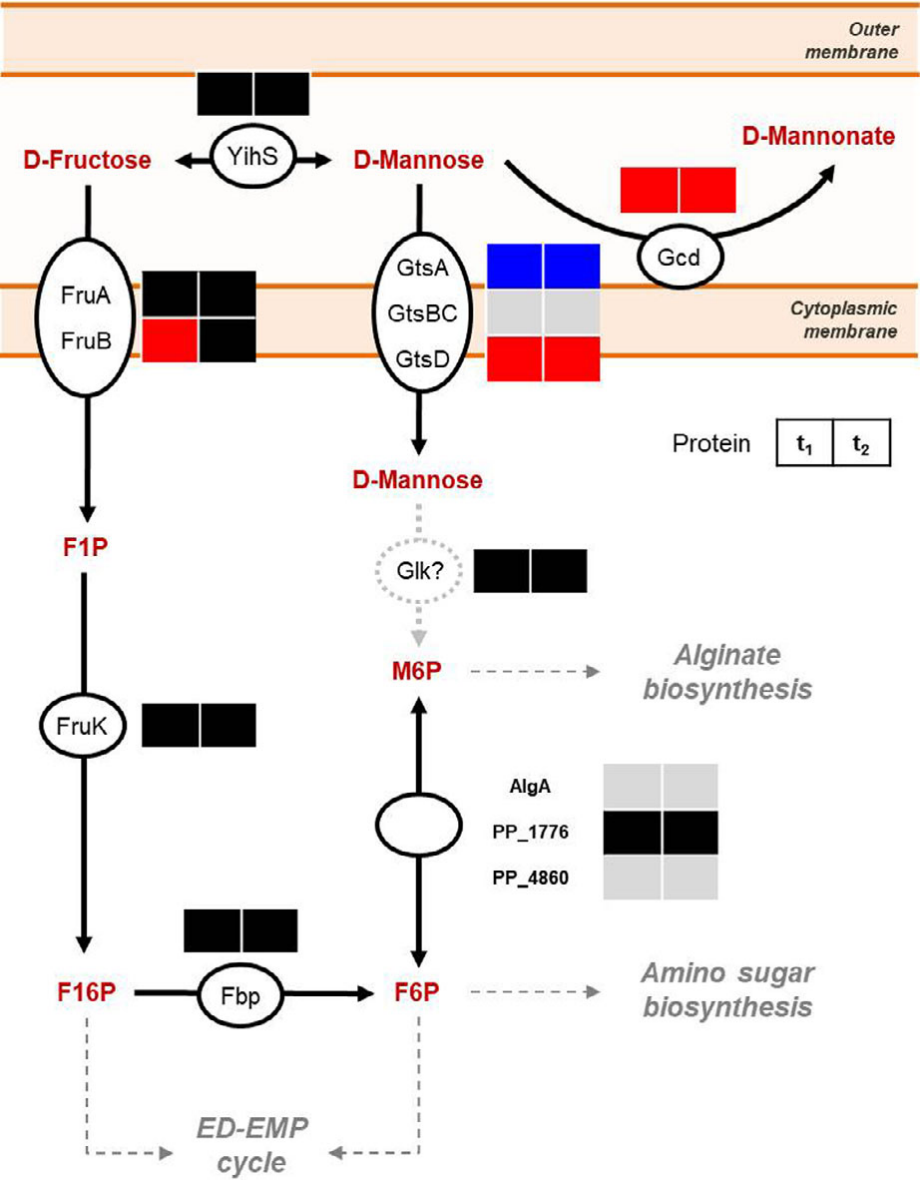
Hypothetical pathway of fructose oxidation, uptake on cytoplasmic membrane, and intracellular metabolism in *P. putida* KT2440. Differential expression analysis of the proteins involved in periplasmic and cytoplasmic fructose metabolism, of samples taken at peak current (t_1_) and 414 h after inoculation (t_2_) during KT2440’s BES cultivation is included. Red colours indicate upregulation, blue indicate down regulation, black indicate insignificant change, grey indicate FC not determined; F1P, fructose 1‐phosphate; M6P, mannose 6‐phosphate; F16P, fructose‐1,6‐bisphosphate; F6P, fructose 6‐phosphate.

The expression of proteins from the peripheral oxidation pathway, glycolytic pathways of the ED–EMP cycle, substrate‐level phosphorylation, pyruvate dehydrogenase complex, tricarboxylic acid (TCA) cycle, glyoxylate shunt and several anaplerotic reactions associated with the central carbon metabolism in KT2440 were analszed in detail (Fig. 3, Table S1–S3). Overall, protein expression tended to decrease over time; however, upregulation of protein in the central carbon metabolism was found almost exclusively during BES experiment (especially at peak current) in contrary to the open‐circuit experiment, where the majority of protein from the central carbon metabolism did not show a significant increase in expression (Fig. 3). While many enzymes of glycolysis were downregulated during BES cultivation, the majority of the proteins from the lower central carbon metabolism (i.e., pathways that follow the metabolite glyceraldehyde 3‐phosphate) were increased in expression level. More specifically, the TCA cycle, was upregulated, or made no significant change during the BES cultivation, especially at peak current, with a few exceptions such as phosphoglycerate kinase (Pgk), isocitrate dehydrogenase (Idh), malate synthase (GlcB) and malic enzyme B (MaeB). Proteins responsible for fructose metabolism were found to be upregulated or have no significant change (Fig. 4, Fig. S8). Surprisingly, the Gcd was the only protein from the peripheral oxidation pathway that was found to be upregulated at both t_1_ and t_2_ (Fig. 3), and was one of the only two peripheral oxidation pathway proteins detected in all of our samples; the other was the cytoplasmic gluconate kinase, GnuK, which did show significant increase at neither t_1_ nor t_2_.

## Discussion

As a saprotrophic, rhizosphere bacterium, *P. putida* might encounter many different plant‐derived carbohydrates, including d‐glucose, d‐galactose and l‐arabinose, which are found predominantly in cellulosic and hemicellulosic hydrolysates, and lignocellulosic biomasses (Derrien *et al*., 2004; Peabody *et al*., 2019; Wang *et al*., 2019). Some of these sugars were not taken up for intracellular metabolism such as d‐galactose or l‐arabinose, but were rather oxidized in the periplasm in BES (Fig. S2, Fig. 1A). The extracellular metabolites measured by GC‐MS were aldonates, or 2‐ketoaldonates (Fig. S2): the reactions with aldoses were therefore very similar to the reactions with glucose in BES, as well as gluconate (GA) and 2‐ketogluconate (2KGA) were produced (Lai *et al*., 2016b; Yu *et al*., 2018). The current densities gradually increased while using those sugars in BES, comparable to previous observations on glucose. There, upregulation of proteins, particular the membrane dehydrogenase involved in the POC pathway, was found to associate with the current increase (Yu *et al*., 2018). This could well‐explain the increase of electron discharge with different aldoses since the same metabolic pathway was used. The present study suggests that Gcd of *P. putida* acts with a broad specificity on the C‐1 position of aldoses, while Gad might have a more confined specificity towards aldohexonates. Similar results were found when KT2440 and other *P. putida* strains were fed with d‐xylose (an aldopentose derived from hemicellulose), which was oxidized to the dead‐end product d‐xylonate (Meijnen *et al*., 2008; Meijnen *et al*., 2009; Bator *et al*., 2019). It is possible that, in its natural habitats where various sugars are present, this feature benefits *P. putida* by generating additional energy when oxygen is present as the terminal electron acceptor through the partial oxidation of sugars that cannot be further metabolized. This finding offers a potential solution for the bioproduction of different value‐added sugar acids and their lactones, while employing a single catalytic system.

In this particular work, however, utilizing aldose sugars poses a challenge for unravelling cytoplasmic metabolism of KT2440 under the BES conditions, since (i) it has been demonstrated that, compared to aerobic conditions, the carbon flux was redirected toward POC, and so far, (ii) there is a lack of evidence of strain KT2440 utilizing sugar acids other than GA and 2KGA, or carbohydrates other than glucose/mannose, fructose and ribose, for growth. But in our opinion, achieving cytoplasmic metabolism is necessary to pinpoint the limitation in central carbon metabolism under BES conditions. For this reason, this study focused on fructose as the substrate for the BES cultivation. Fructose is a hexose with a keto‐function at the C‐2 position, which was thought to make it inaccessible for oxidation by Gcd. Instead, fructose is imported into the cytoplasm via a phosphoenolpyruvate‐dependent phosphotransferase system. This PTS transporter is encoded by the *fruBKA* operon in *P. putida* KT2440 (Sawyer *et al*., 1977; Chavarría *et al*., 2012; Chavarría *et al*., 2016). It is also worth noting that, under aerobic conditions, the cells channel around 48% of the carbon through EMP and PP pathways when fed with fructose, compared to only ~ 4% with glucose (Chavarría et al., 2012); for our study, we anticipated that this could help overcome the previously observed decrease in NADPH/NADP^+^ ratio in anaerobic *P. putida* culture by improving NADPH fluxes (Nikel *et al*., 2020). Our proteomic analysis suggests that intracellular uptake of fructose was taking place, as demonstrated by the expression of the *fruBKA* operon and the putative glyceraldehyde 3‐phosphate dehydrogenase (GapDH) PP_3443 in the BES experiment (Fig. 2). Both loci are expressed under the modulation of fructose 1‐phosphate – a cytoplasmic metabolite that can only be produced via the fructose PTS activity (Chavarría *et al*., 2014; Chavarría *et al*., 2016). At the same time, periplasmic oxidation activity was observed occurring during BES cultivation: fructose was unexpectedly metabolized to mannose, which then could be oxidized by Gcd. Since abiotic control experiments indicated that fructose was not abiotically converted to other sugars, in addition to our analysis of extracellular metabolites, we hypothesized that fructose has been isomerized into mannose by the enzyme aldose‐ketose isomerase (YihS), whose gene locates next to (i.e., 110 base pairs upstream of) the glucose/mannose ABC transporter operon *gtsABCD* (Nelson *et al*., 2002; Belda *et al*., 2016). YihS is found widely distributed in bacteria, and possibly sharing a similar mechanism of action as YihS in *Escherichia coli* and *Salmonella enterica* (Itoh *et al*., 2008). While YihS was not specifically upregulated, we found the protein in almost all replicates under BES conditions. It readily interconverts fructose and mannose, which can be further taken up by either PTS or ABC transporter system (Fig. 4). Mannose, as an aldose, could also be subjected to POC activity, in which an aldonate (i.e., mannonate) was subsequently produced (Fig. 2, Fig. S5). We observed a significant increase in Gcd expression throughout the BES cultivation of KT2440 (Fig. 3, Table S1, Table S2), which might have helped maintain mannonate production. The reason behind its upregulation, nonetheless, remains unknown; as Gcd expression and its activity depend on several factors, such as the availability of sugar, oxygen, cAMP, soluble phosphate and co‐factor pyrroloquinoline quinone (Yamada *et al*., 1993; An and Moe, 2016; Zeng *et al*., 2016), the explanation for this behaviour, hence, might not be straight forward. No protein from the Gad complex and 2‐ketogluconate uptake pathway was detected from our proteome analysis in neither BES nor open‐circuit control experiments, explaining the lack of the corresponding 2‐ketoaldonate production in strain KT2440. This is unlike the case of d‐galactose where both galactonate and 2‐ketogalactonate were detected (Fig. S2). It is possible that, nonetheless, the cells of the galactose culture only expressed Gad‐like behaviour as a result of pre‐culturing in glucose. The absence of these proteins, or any other protein, in the dataset, nevertheless, should not be construed as a non‐expression due to the inherent limitation of the dynamic exclusion filtering (Zhang *et al*., 2006) used in our shot‐gun proteomics. Even if the Gad complex was present, it still might not be able to further oxidize mannonate due to the incorrect stereogenic centre at the C‐2 position compared to gluconate. Finally, the higher ATP/ADP ratio associated with the presence of the Gcd, once again, might indicate that this pathway served as an energy source rather than a carbon uptake route for the cell.

Based on the insignificant differences in pyruvate and acetate production (Table 1), the net fluxes in the intracellular carbon metabolism between the KT2440 and KT2440Δ*gcd* strains appeared unchanged. When comparing our results to the BES culture of KT2440 on glucose (Yu *et al*., 2018), pyruvate accumulation was similar in both cases, whereas acetate production yield from fructose was 2.5‐times lower. The change in metabolism was indicated more clearly in the shift in NADPH/NADP^+^ ratio at the peak current of the BES cultivation. In the previous observation with BES cultivation of *P. putida* F1 with glucose, the NADP pool was found to be more reduced, pointing at a limitation in NADPH‐generating pathways (Lai *et al*., 2016b). This study with fructose, however, revealed the complete opposite phenomenon as NADPH/NADP^+^ increased throughout the BES experiment (Table 2, Fig. 4). One possible contributing factor was that 2‐ketogluconate 6‐phosphate reductase (KguD), which reduces 2‐ketogluconate 6‐phosphate to 6‐phosphogluconate by consuming NADPH, might not be expressed, or, inactive when fructose was the substrate. The influence of this reaction on the overall NADP pool of the cell, remains to be elucidated. Meanwhile, expression analysis of proteins involved in NADPH regeneration in the central carbon metabolism (Zwf, GntZ, GapDH, Icd and Idh, Fig. 3D) and anaplerotic pathways (MaeB, GltBD, Fig. 3D;) revealed that these proteins were either upregulated or remained the same when peak current was reached. On the other hand, even though proteins involved in NADH regeneration (GapDH, 2‐keto glutarate dehydrogenase complex, Mdh, Mqo, GdhB, Fig. 3D) were found to be upregulated, the NAD pool became much more oxidized, in contrast to a more reduced NADH/NAD^+^ ratio across different anaerobic setups in strain F1 on glucose (Table 2). As the expression of many of these enzymes is controlled by the global regulators modulated by intracellular redox state in strictly aerobic and facultatively anaerobic bacteria (Park *et al*., 1997; Perrenoud and Sauer, 2005; Vemuri *et al*., 2006), the changes in protein expression of the TCA cycle enzymes might have been the result of cell response to the shifts in NAD(P)H/NAD(P)^+^ ratios. Conversely, the proteomic data did not reflect enzymatic activities; thus, the full extent of protein expression’s impact on internal redox ratios remained to be investigated. Interestingly, many anaplerotic, amino‐acid‐catabolizing enzymes were also found upregulated, resembling KT2440 cells under glucose starvation (Ankenbauer *et al*., 2020), suggesting that the cytoplasmic carbon flux from upstream fructose metabolism might still be insufficient to functionalize the TCA cycle. This result implied that it would take more than just changing substrate in order to achieve cell growth under anaerobic condition. Nonetheless, the systematic upregulation of proteins of the central carbon metabolism pathways proved that the combination of an available pool of electron acceptor (in our case, oxidized ferricyanide) and a substrate that the cell could take up (fructose) would be sufficient to maintain cellular activity for a period of time, at least at the protein expression level.

Based on the analyses above, we anticipate that targeting transcriptional regulators of central carbon metabolism is one possible engineering strategy for improving cultivation under BES conditions in the future, as the intracellular uptake of sugars remained rather low due to the shift of carbon flux toward energy‐generating pathway in the periplasm. In this scenario, one promising protein is the global transcriptional regulator HexR, which negatively regulates the expression of *gap‐1* gene, the *edd‐glk‐gltR‐2* and *zwf‐pgl‐eda* operons under modulation of KDPG (Table S3). The protein expression pattern of the ED‐EMP cycle in our study suggested that repression by HexR might have been taking place (Fig. 3D). A similar approach, which included a HexR knockout mutation, has been previously attempted to boost a highly NADPH‐demanding *para*‐hydroxy benzoate production in KT2440 (Yu *et al*., 2016). Additional mutation of negative regulators, such as PtxS, which regulates 2KGA metabolism (del Castillo *et al*., 2008; Daddaoua *et al*., 2010; Udaondo *et al*., 2018), or Cra, might also add to the alleviation of limited intracellular carbon flux of KT2440 in the case of anoxic catabolism of glucose and fructose, respectively. Nevertheless, improving the carbon flux would only be a part of the puzzle: an *in‐silico* approach by Kampers *et al*. (2021) revealed that genetic and metabolic engineering to enable a facultatively anaerobic lifestyle in KT2440 was a much more elaborate process than previously thought. Thus, substitution of oxygen‐dependent reactions to acclimate the cell to anoxic environment of the BES should also be taken into consideration.

In summary, our study has shown the versatility of *P*. *putida* in metabolizing carbohydrates under BES conditions. While we did not succeed in achieving anaerobic growth, this study describes the physiological changes during anaerobic fructose metabolism and points to future optimization strategies. Implementing the recently published oxygen‐independent anabolic reactions (Kampers *et al*., 2019) and further works on de‐regulation of the central carbon metabolism might be able to finally allow anaerobic metabolism and growth in *P. putida*.

## Experimental procedures

### Bacterial strains and growth condition of pre‐cultures

*P. putida* KT2440 was ordered from DSMZ, Germany, and its Gcd knockout mutant was previously constructed by Sánchez‐Pascuala *et al*. (2019). Cryostocks were initially activated at 30°C in lysogeny broth (LB) before pre‐culture cultivation. The bacterial pre‐cultures for BES experiment were prepared in defined mineral medium (DM9) using either d‐(+)‐glucose (for testing different aldoses) or d‐(‐)‐fructose (for testing fructose) as carbon source (final concentration of 5 g L^‐1^) as described elsewhere (Lai *et al*., 2016b). Chemicals were ACS grade with ≥ 98% purity. All solutions were prepared in ultrapure H_2_O (18.2 MΩ⋅cm, 2 ppb TOC).

### BES setup and operation

The BES reactor setup and operational procedures were previously described by Lai *et al*. (2019). Each reactor contained 2 g l^‐1^ of tested monosaccharide. Pre‐cultured KT2440 strains in glucose were added as inocula if an aldose was tested, while pre‐cultures in fructose were used solely for experiments with fructose. The optical density of the cell population was analysed by a Libra S12 UV–Vis spectrophotometer (Biochrom Ltd., Cambourne, Cambridge, UK) using optical density at 600 nm (OD_600_) and converted to cell dry weight (CDW) by following the empirically determined conversion factor: CDW (g l^‐1^) ≈ 0.476 × OD_600_, with the coefficient assumed as the same for all strains. The amount of electrons (in mmol) released from the cellular catabolism and harvested by the anode were calculated as following: n_e‐_ = 1000 × Q/*F*, with Q, the cumulative charge (in C). The value of Faraday’s constant (*F*) was approximately 96485.3 C mol^‐1^. The presence of both fructose and ferricyanide generated a high background current, which should be subtracted from the signals obtained in biotic experiments.

### Exo‐metabolites identification and quantification using GC‐MS and HPLC

Extracellular metabolites were quantified using high‐performance liquid chromatography (HPLC). The BES cell cultures were centrifuged at 4°C, 17 000 g for 10 min and the supernatants were collected and analysed using the protocols described before (Lai *et al,l*., 2016a). The quantitative calibration of d‐mannonate was done by hydrolyzing the d‐mannono‐1,4‐lactone (abcr GmbH, Karlsruhe, Germany).

Unknown extracellular metabolites were identified by a gas chromatography–mass spectrometry (GC‐MS) system (ISQ™ LT Single Quadrupole, Thermo Scientific, Waltham, MA, USA) equipped with an off‐axis discrete dynode electron multiplier and electrometer (Dynamax^TM^ XR detection system), a TR‐5MS GC Column (30 m × 0.25 mm; Thermo Scientific), operated with He as the carrier gas. Prior to analysis, 15 nmol of adonitol was added in 5‐10 µl of culture supernatant as internal standard and then blow‐dried with pure N_2_ gas. The dried sample was derivatized with 50 µl of MOX reagent (2% Methoxylamine.HCl in pyridine; Thermo Scientific) at 40°C for 1 h, followed by 50 µl of SILYL‐991 (N,O‐Bis(trimethylsilyl)trifluoroacetamide:chlorotrimethylsilane 99:1, Macherey‐Nagel, Düren, NRW, Germany) at 80°C for 2 h. The derivatized sample was injected in splitless mode. The temperature profile setting was as follows: 60°C (1 min), 60–270°C (8.5°C min^‐1^), and 270–325°C (70°C min^‐1^). Products were validated using external standard compounds and ready‐made MS libraries from the National Institute of Standard and Technology (NIST, Gaithersburg, MD, USA).

### Measurement of the intracellular energy and redox co‐factors

ATP/ADP and the NAD(P)H/NAD(P)^+^ ratios were measured using protocol similar to Lai *et al*. (2016b). Approximately 50 mg of cells were prepared for the following assays by washing with a cold phosphate buffer (pH 7.75, pre‐cooled to 4°C) and collected by centrifugation at 4°C, 17 000 g for 1 min. Metabolism was quenched by rapid freezing of cell pellets in liquid N_2_. Adenylate molecules were extracted by adding 250 µl ice‐cold 5% (w/v) trichloroacetic acid (TCA) in 4 mM EDTA to a 250 µl cell re‐suspension in cold phosphate buffer. Conversion of ADP to ATP was carried out using pyruvate kinase (Merck, Darmstadt, Germany). Quantification of ATP using ATP determination kit, PRO (Biaffin GmbH & Co KG, Kassel, Germany) and inhibition coefficient of TCA on the assay were previously reported (Lai *et al*., 2016b). Commercial assay kits of NADH and NADPH (K338 & K349, BioVision Inc., Milpitas, California) were used and the extraction and quantification of respective co‐factors were conducted following the manufacture’s description.

### Proteome analysis using mass spectrometry

Approximately 200 µg cell pellets were harvested by centrifugation (4°C, 17 000 g, 10 min) at inoculation time (t_0_), at the time of peak current (t_1_), and 414 h after inoculation (t_2_). Cells were washed in 50 mM ammonium bicarbonate and disrupted with 3 cycles of flash freezing‐thawing in liquid N_2_. Glyceraldehyde 3‐phosphate dehydrogenase from *Staphylococcus aureus* Mrsa252 was added to each sample as the internal standard. The samples were then reduced with 10 mM dithiothreitol in 50 mM ammonium bicarbonate for 1 h at 30°C. Subsequent alkylation of cysteine residues was performed using 100 mM iodoacetamide in 50 mM ammonium bicarbonate for 1 h at room temperature in the dark. Trypsin digestion was carried out by incubation overnight at 37°C with slight shaking using a final concentration of 0.1 µg µl^‐1^ sequencing‐grade modified trypsin (Promega Co., Madison, WI, United States). Peptide samples were desalted using C18 Zip Tip columns (Merck Millipore, Darmstadt, Germany). Each biological sample was divided into three technical replicates, prior to analysis via nano‐liquid chromatography–tandem mass spectrometry (nLC‐MS/MS) using an Orbitrap Fusion Tribrid mass spectrometer (Thermo Scientific) equipped with a nanoLC system (Dionex Ultimate 3000RSLC; Thermo Scientific). Peptides were separated on a Acclaim PepMap100 C18 column (250 mm × 0.075 mm, 3 µm particle size, Thermo Scientific) at a flow rate of 0.3 μl min^−1^ by applying the following settings with eluent A (0.1% formic acid in water) and eluent B (80% (v/v) acetonitrile and 0.1% formic acid in water): column equilibration for 1 min at 4% B, then followed by a gradual increase to 10% B within 4 min, to 35% B within 95 min, to 55% B within 20 min, to 90% B within 10 min, and finally hold at 90% B for 5 min. The operational method for the mass spectrometer was specified elsewhere (Seidel *et al*., 2018).

Protein identification was conducted by Proteome Discoverer (v2.4, Thermo Fisher Scientific) using the SequestHT as a search engine with the UniProt database of *P. putida* KT2440 with the following settings: cleavage enzyme trypsin, peptide length of 6‐144 residues, allowing up to two missed cleavages, precursor mass tolerance and fragment mass tolerance were set to 3 ppm and 0.1 Da, respectively. Oxidation of methionine residues and carbamidomethylation of cysteine residues were selected as dynamic modification and static modification, respectively. The false discovery rate of identified peptide sequences was kept to < 0.01 using the Percolator node. The abundance of proteins and peptides was calculated by label‐free quantification based on area counts using the Minora node implemented in Proteome Discoverer. The relative protein abundance or relative peptide abundance was normalized to the abundance of the internal standard. The abundance of each protein at each sampling point was calculated as the average of the abundances in all replicates. Log fold change (FC) of protein expression during the cultivation was calculated as the fold change of the abundance at a time point (*t_i_*, *i* = 1 or 2) versus the abundance at time *t*_0_:

$$\log_{2}\left(FC\right)=\log_{2}\left(\frac{Abundance(t_{i})}{Abundance(t_{0})}\right).$$

One‐tailed paired *t*‐test was used for computing *P*‐values of two dependent sets of abundances. Volcano plots were generated from significance (‐log_10_(*P*‐value)) against log_2_(FC).

## Funding Information

We thank Dr. Pablo I. Nikel at Novo Nordisk Foundation Center for Biosustainability, Denmark, for the provision of the *gcd* knockout strain, Benjamin Scheer (UFZ) for technical support of the nLC‐MS/MS, Caroline Ruhl and Ron Stauder (UFZ) for valuable help and discussion regarding the analytics. AVN acknowledges scholarship support from the Helmholtz Center for Environmental Research – UFZ within the STROMER PhD College. Protein mass spectrometry was done at the Centre for Chemical Microscopy (ProVIS) at the Helmholtz Centre for Environmental Research, which is supported by European regional development funds (EFRE—Europe Funds Saxony) and the Helmholtz Association.

## Conflict of interest

The authors declare no commercial or financial relationships that could be construed as a potential conflict of interest.

## Author contributions

AVN designed, conducted the experiments, and analysed the data under the supervision of BL and JOK. LA supervised the proteomics experiments and helped with data analysis. All authors contributed to scientific discussion, as well as drafting and editing the manuscript.

## Supporting information


**Fig. S1**. Central carbon metabolism in *P. putida* KT2440. Each green box represents an intermediate metabolite of the pathway. All reactions occur before/toward the node GA3P are considered upper carbon metabolism, while the ones after GA3P are lower carbon metabolism. D‐Fru, fructose; F1P, fructose 1‐phosphate; D‐Man, mannose; M6P, mannose 6‐phosphate; D‐Glu, glucose; G6P, glucose 6‐phosphate; F6P, fructose 6‐phosphate; F16P, fructose 1,6‐bisphosphate; DHAP, dihydroxyacetone phosphate; RL5P, ribulose 5‐phosphate; GA, gluconate; 2KGA, 2‐ketogluconate; 6PKGA, 6‐phospho 2‐ketogluconate; 6PG, 6‐phosphogluconate; KDPG, 2‐dehydro‐3‐deoxy‐phosphogluconate; GA3P, glyceraldehyde 3‐phosphate; 13BPG, 1,3‐biphosphoglycerate; 3PG, 3‐phosphoglycerate; 2PG, 2‐phosphoglycerate; PEP, phosphoenolpyruvate; Pyr, pyruvate; Ace‐CoA, acetyl coenzyme A; Cit, citrate; Isocit, isocitrate; 2KG, 2‐ketoglutarate; Suc‐CoA, Succinyl coenzyme A; Suc, succinate; Fum, fumarate; Mal, (D,L‐) malate; Oxa, oxaloacetate; Glyox, glyoxylate.
**Fig. S2**. Aerobic growth of *P. putida* KT2440 with different monosaccharides as substrate. (**A**) Growth curve of KT2440 from single colony in DM9 medium with different sugars as carbon source; data are averages of biological triplicates, error bar on each data point represents the standard deviation of sample (*n* = 3). Sugar concentration and optical density of KT2440 when cultivated in (**B**) D‐galactose and (**C**) L‐arabinose; data are averages of biological triplicates, error bar on each data point represents the standard deviation of sample (*n* = 3). GC‐MS analysis of extracellular metabolites at the beginning (black line), one day after inoculation (blue line) and at the end (magenta line) of the cultures in (**D**) D‐galactose and (**E**) L‐arabinose against a negative control (green line); peak **1**, adonitol‐5TMS (internal standard), **2a,b**, galactose‐1MOX‐5TMS; **3**, galactonic acid‐6TMS; **4**, galactono‐1,4‐lactone‐4TMS; **5a,b**, 2‐keto galactonic acid‐1MOX‐5TMS; **6**, 2‐keto galactonic acid‐5TMS; **7**, arabinose‐1MOX‐4TMS; **8**, arabinonic acid‐5TMS.
**Fig. S3**. Optical densities (**A, C**) and absolute sugar contents (**B, D**) of KT2440 and KT2440Δ*gcd* cultures in BES with various aldoses (*n* = 1 for each sugar).
**Fig. S4**. GC‐MS analysis of extracellular metabolite composition of KT2440 culture in (**A**) D‐galactose, (**B**) L‐arabinose, and (**C**) D‐ribose approximately 200 hours after inoculation (black line) and at the end (blue line) of the BES experiments. Peak **1**, adonitol‐5TMS (internal standard); **2a,b**, galactose‐1MOX‐5TMS; **3**, galactonic acid‐6TMS; **4**, galactono‐1,4‐lactone‐4TMS; **5a**,**b**, 2‐keto galactonic acid‐1MOX‐5TMS; **6**, 2‐keto galactonic acid‐5TMS; **7**, arabinose‐1MOX‐4TMS; **8**, arabinonic acid‐5TMS; **9**, ribose‐1MOX‐4TMS; **10**, ribonic acid‐5TMS.
**Fig. S5**. GC‐MS standard compound identification (**A**, green, adonitol; red, D‐fructose; purple, D‐mannose; orange, D‐mannonate) and analysis of extracellular metabolite composition of (**B**) KT2440’s and (**C**) KT2440Δ*gcd*’s BES cultures at t = 2 h (black line), t = 146 h (blue line), t = 340 h (magenta line) and t = 480 h (brown line). Peak **1**, adonitol‐5TMS (internal standard); **2a**,**b**, fructofuranose‐5TMS; **3a**,**b**, fructose‐1MOX‐5TMS; **4**, mannonic acid‐6TMS; **5**, mannono‐1,4‐lactone‐4TMS; **6a**,**b**, mannose‐1MOX‐5TMS.
**Fig. S6**. Adsorption of *P. putida* (A) KT2440’s and (B) KT2440Δ*gcd*’s biomasses on anode’s surface.
**Fig. S7**. NADPH/NADP^+^ and NADH/NAD^+^ ratios in KT2440 cell extracts over the course of their BES cultivation. Data are average of three biological replicates; error bars represent the standard deviation of sample (*n* = 3).
**Fig. S8**. (A) Current density of KT2440 cultures (*n* = 3) observed during BES cultivation with 2 g L^‐1^ fructose and three sampling points for proteomics; KT2440 cultures (*n* = 2) with open‐circuit setting were simultaneously sampled at the same time points, using absorbance of ferricyanide as indicator of substrate oxidation. Data are averages of biological replicates; gray area and error bars represent the standard deviations of sample. Volcano plots of KT2440’s proteome during BES cultivation with fructose at t_1_ versus t_0_ (B) and t_2_ versus t_0_ (C), and volcano plots of KT2440’s proteome during open‐circuit cultivation with fructose at t_1_ versus t_0_ (D) and t_2_ versus t_0_ (E).
**Table S1**. Relative protein abundance of *P. putida* KT2440 during BES (*n* = 3) and open‐circuit (*n* = 2) cultivation in fructose.
**Table S2**. Fold change and significance of central carbon metabolism’s protein expression changes in *P. putida* KT2440 during BES cultivation in fructose (*n* = 3).
**Table S3**. Fold change and significance of central carbon metabolism’s protein expression changes in *P. putida* KT2440 during open‐circuit cultivation in fructose (*n* = 2).
**Table S4**. Differential expression analysis of selected regulatory proteins of the CCM in *P.putida* KT2440 during BES cultivation in fructose.
