# Supplementary material for: The anoxic electrode‐driven fructose catabolism of Pseudomonas putida KT2440
Source: Microb Biotechnol. 2021 Jun 11;14(4):1784–96. doi: 10.1111/1751-7915.13862 (PMC8313287; doi:10.1111/1751-7915.13862)
Supplement: Supplementary file 1 — Fig. S1. Central carbon metabolism in P. putida KT2440. Each green box represents an intermediate metabolite of the pathway. All reactions occur before/toward the node GA3P are considered upper carbon metabolism, while the ones after GA3P are lower carbon metabolism. D‐Fru, fructose; F1P, fructose 1‐phosphate; D‐Man, mannose; M6P, mannose 6‐phosphate; D‐Glu, glucose; G6P, glucose 6‐phosphate; F6P, fructose 6‐phosphate; F16P, fructose 1,6‐bisphosphate; DHAP, dihydroxyacetone phosphate; RL5P, ribulose 5‐phosphate; GA, gluconate; 2KGA, 2‐ketogluconate; 6PKGA, 6‐phospho 2‐ketogluconate; 6PG, 6‐phosphogluconate; KDPG, 2‐dehydro‐3‐deoxy‐phosphogluconate; GA3P, glyceraldehyde 3‐phosphate; 13BPG, 1,3‐biphosphoglycerate; 3PG, 3‐phosphoglycerate; 2PG, 2‐phosphoglycerate; PEP, phosphoenolpyruvate; Pyr, pyruvate; Ace‐CoA, acetyl coenzyme A; Cit, citrate; Isocit, isocitrate; 2KG, 2‐ketoglutarate; Suc‐CoA, Succinyl coenzyme A; Suc, succinate; Fum, fumarate; Mal, (D,L‐) malate; Oxa, oxaloacetate; Glyox, glyoxylate. Fig. S2. Aerobic growth of P. putida KT2440 with different monosaccharides as substrate. (A) Growth curve of KT2440 from single colony in DM9 medium with different sugars as carbon source; data are averages of biological triplicates, error bar on each data point represents the standard deviation of sample (n = 3). Sugar concentration and optical density of KT2440 when cultivated in (B) D‐galactose and (C) L‐arabinose; data are averages of biological triplicates, error bar on each data point represents the standard deviation of sample (n = 3). GC‐MS analysis of extracellular metabolites at the beginning (black line), one day after inoculation (blue line) and at the end (magenta line) of the cultures in (D) D‐galactose and (E) L‐arabinose against a negative control (green line); peak 1, adonitol‐5TMS (internal standard), 2a,b, galactose‐1MOX‐5TMS; 3, galactonic acid‐6TMS; 4, galactono‐1,4‐lactone‐4TMS; 5a,b, 2‐keto galactonic acid‐1MOX‐5TMS; 6, 2‐keto galactonic acid‐5TMS [file MBT2-14-1784-s001.docx]

# Suppplementary figures and tables

**The anoxic electrode-driven fructose catabolism of *Pseudomonas putida* KT2440**

by Anh Vu Nguyen^1^, Bin Lai^1^, Lorenz Adrian^2,3^, and Jens O. Krömer^1^*

^1^ Department of Solar Materials, Helmholtz Centre for Environmental Research - UFZ, Leipzig, Germany

^2^ Department of Environmental Biotechnology, Helmholtz Centre for Environmental Research - UFZ, Leipzig, Germany

^3^ Chair of Geobiotechnology, Technische Universität Berlin, Berlin, Germany

**Fig. S1.** Central carbon metabolism of *P. putida* KT2440

**Fig. S2.** Aerobic growth and carbohydrate degradation by *P. putida* KT2440 in different sugar substrates

**Fig. S3.** Optical densities and absolute sugar contents of KT2440 and KT2440Δ*gcd* cultures in BES with various aldoses

**Fig. S4.** Identification of extracellular metabolites during BES cultivation in different aldoses using GC-MS

**Fig. S5.** Identification of extracellular metabolites during BES cultivation in fructose using GC-MS

**Fig. S6.** Adsorption of *P. putida* cells on anode’s surface

**Fig. S7.** NAD(P)H/NAD(P)+ ratio of KT2440 cells during BES cultivation in fructose

**Fig. S8.** Sampling points and volcano plots of KT2440’s proteome during BES and open-circuit cultivations

**Table S1.** Relative protein abundance of *P. putida* KT2440 during BES and open-circuit cultivation in fructose

**Table S2.** Fold change and significance of CCM protein expression changes in *P. putida* KT2440 during BES cultivation in fructose

**Table S3.** Fold change and significance of CCM protein expression changes in *P. putida* KT2440 during open-circuit cultivation in fructose

**Table S4.** Differential expression analysis of selected CCM regulatory proteins in *P. putida* KT2440


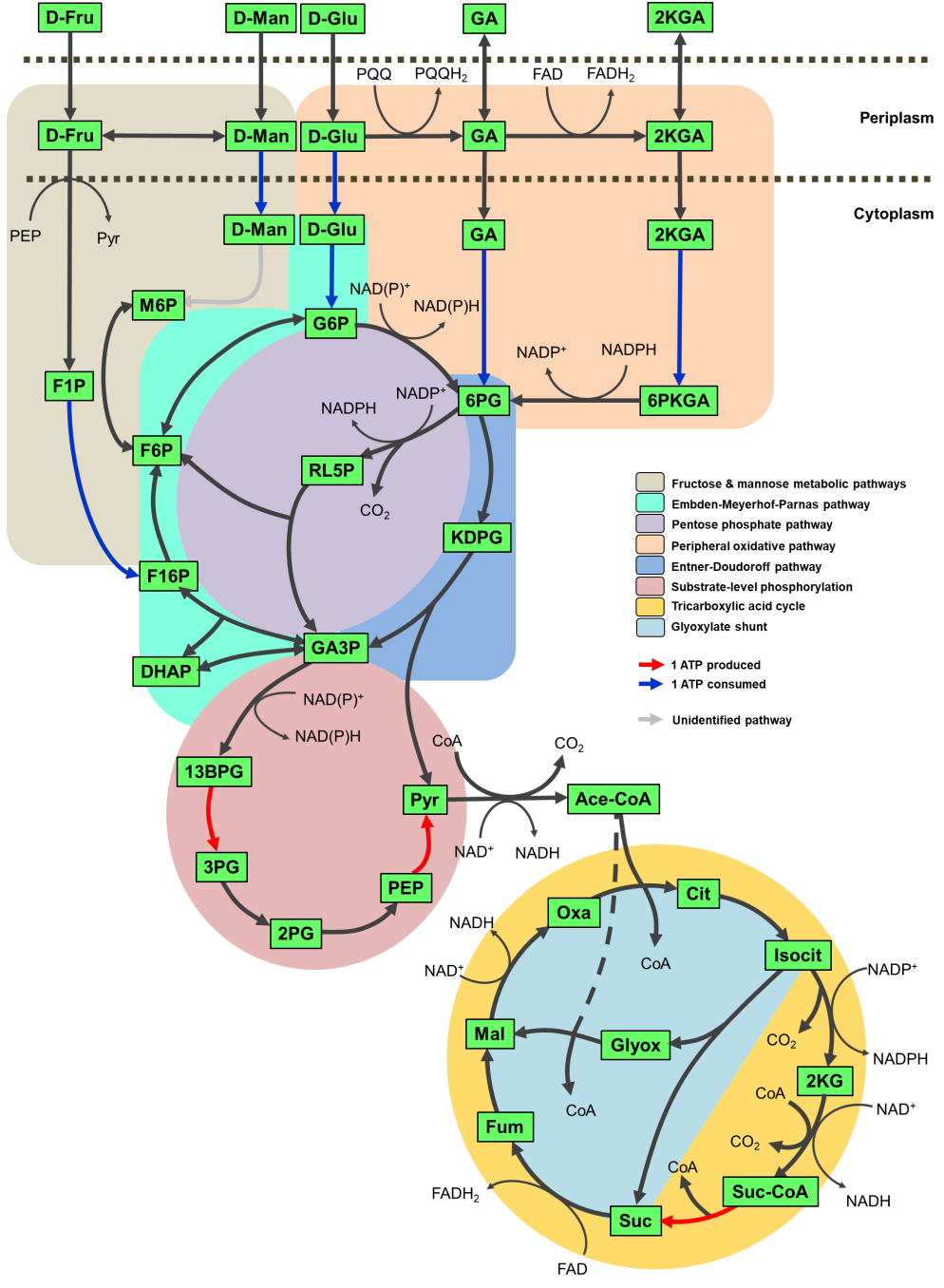


**Fig. S1.** Central carbon metabolism in *P. putida* KT2440. Each green box represents an intermediate metabolite of the pathway. All reactions occur before/toward the node GA3P are considered upper carbon metabolism, while the ones after GA3P are lower carbon metabolism. D-Fru, fructose; F1P, fructose 1-phosphate; D-Man, mannose; M6P, mannose 6-phosphate; D-Glu, glucose; G6P, glucose 6-phosphate; F6P, fructose 6-phosphate; F16P, fructose 1,6-bisphosphate; DHAP, dihydroxyacetone phosphate; RL5P, ribulose 5-phosphate; GA, gluconate; 2KGA, 2-ketogluconate; 6PKGA, 6-phospho 2-ketogluconate; 6PG, 6-phosphogluconate; KDPG, 2-dehydro-3-deoxy-phosphogluconate; GA3P, glyceraldehyde 3-phosphate; 13BPG, 1,3-biphosphoglycerate; 3PG, 3-phosphoglycerate; 2PG, 2-phosphoglycerate; PEP, phosphoenolpyruvate; Pyr, pyruvate; Ace-CoA, acetyl coenzyme A; Cit, citrate; Isocit, isocitrate; 2KG, 2-ketoglutarate; Suc-CoA, Succinyl coenzyme A; Suc, succinate; Fum, fumarate; Mal, (D,L-) malate; Oxa, oxaloacetate; Glyox, glyoxylate.


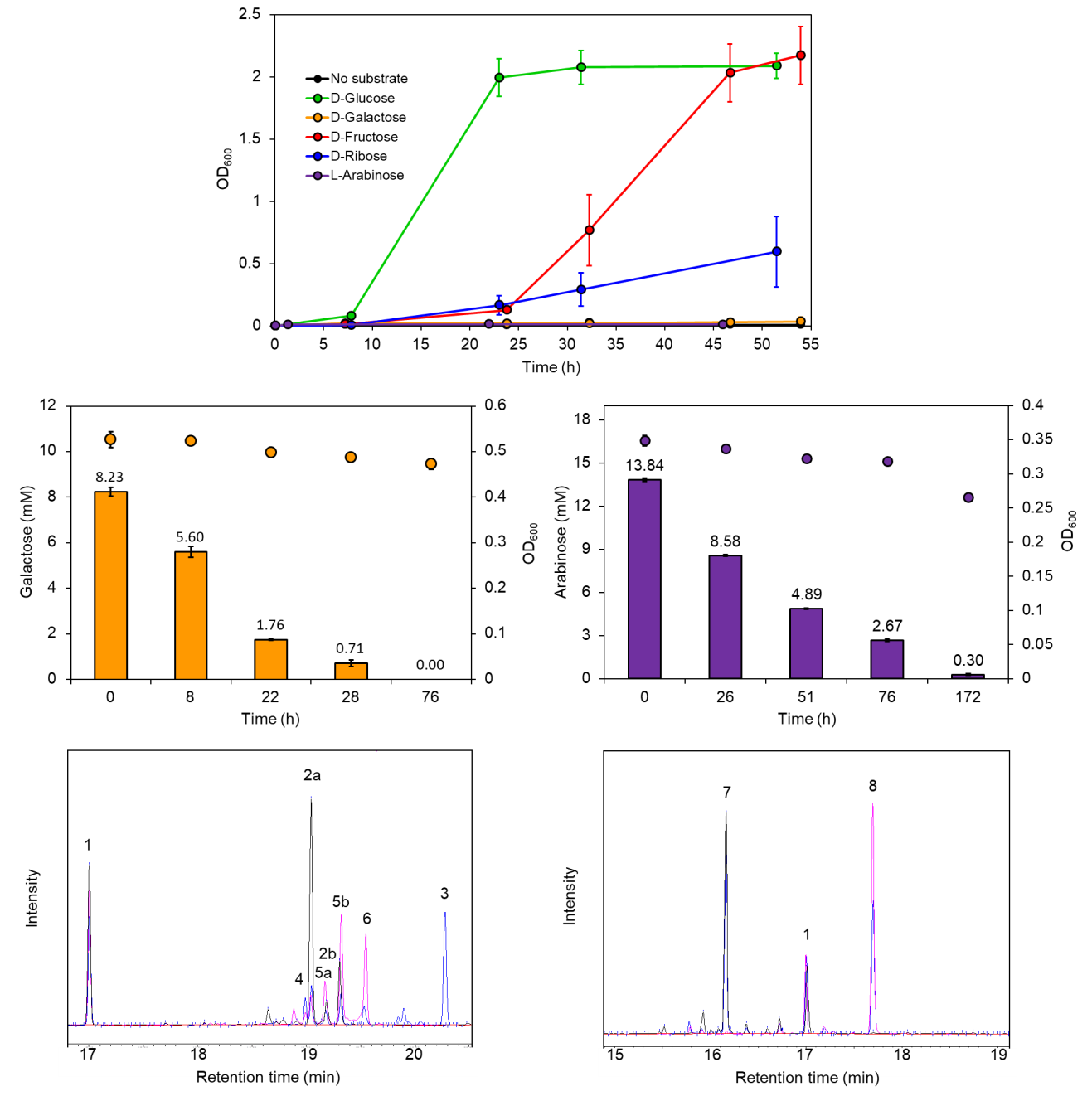


**A**

**B**

**C**

**D**

**E**


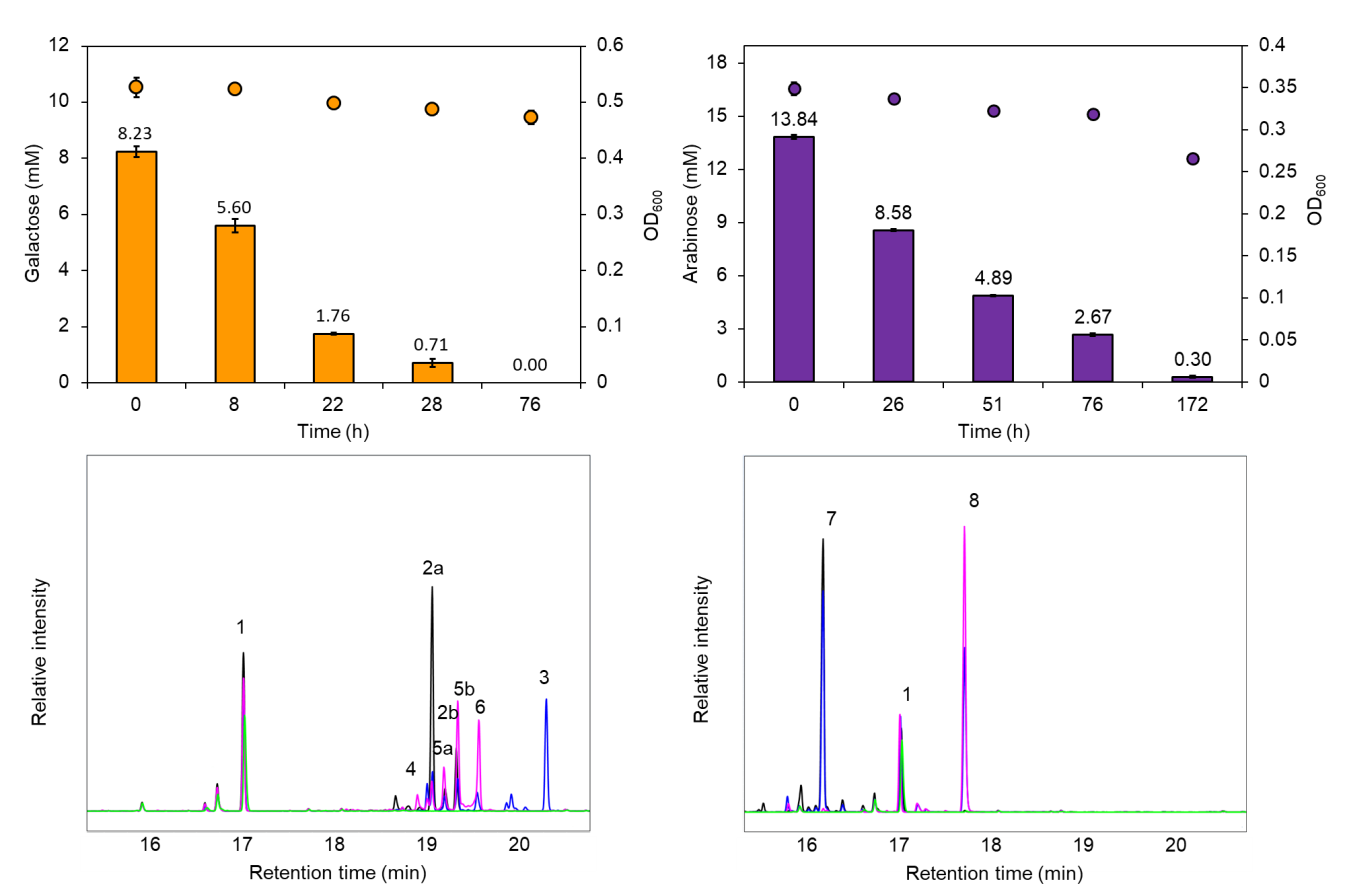


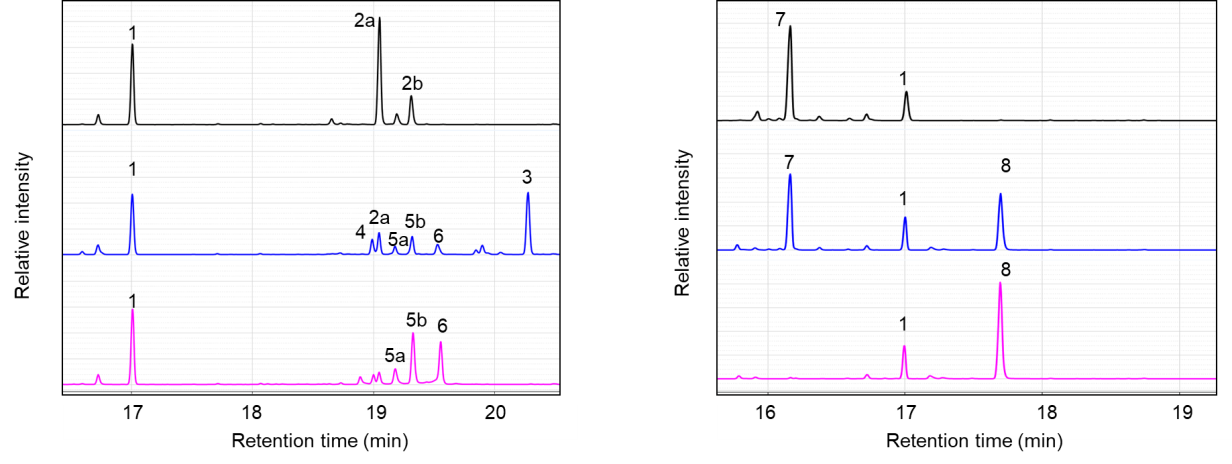


**Fig. S2.** Aerobic growth of *P. putida* KT2440 with different monosaccharides as substrate. (**A**) Growth curve of KT2440 from single colony in DM9 medium with different sugars as carbon source; data are averages of biological triplicates, error bar on each data point represents the standard deviation of sample (n=3). Sugar concentration and optical density of KT2440 when cultivated in (**B**) D-galactose and (**C**) L-arabinose; data are averages of biological triplicates, error bar on each data point represents the standard deviation of sample (n=3). GC-MS analysis of extracellular metabolites at the beginning (black line), one day after inoculation (blue line) and at the end (magenta line) of the cultures in (**D**) D-galactose and (**E**) L-arabinose against a negative control (green line); peak **1**, adonitol-5TMS (internal standard), **2a,b**, galactose-1MOX-5TMS; **3**, galactonic acid-6TMS; **4**, galactono-1,4-lactone-4TMS; **5a,b**, 2-keto galactonic acid-1MOX-5TMS; **6**, 2-keto galactonic acid-5TMS; **7**, arabinose-1MOX-4TMS; **8**, arabinonic acid-5TMS.


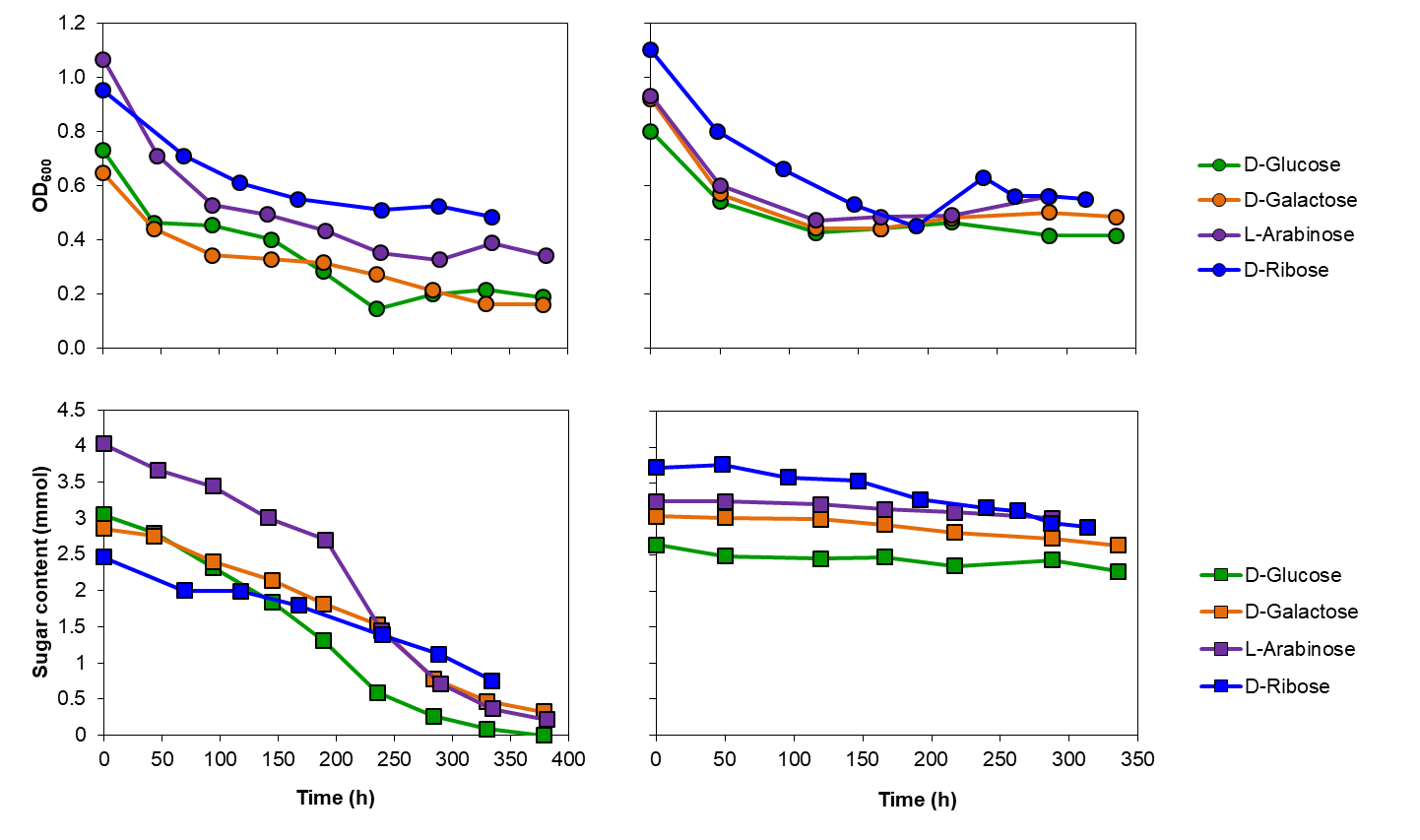


**A**

**CA**

**B**

**DA**

**KT2440**

**KT2440Δ*gcd***

**Fig. S3.** Optical densities (**A, C**) and absolute sugar contents (**B, D**) of KT2440 and KT2440Δ*gcd* cultures in BES with various aldoses (n=1 for each sugar).


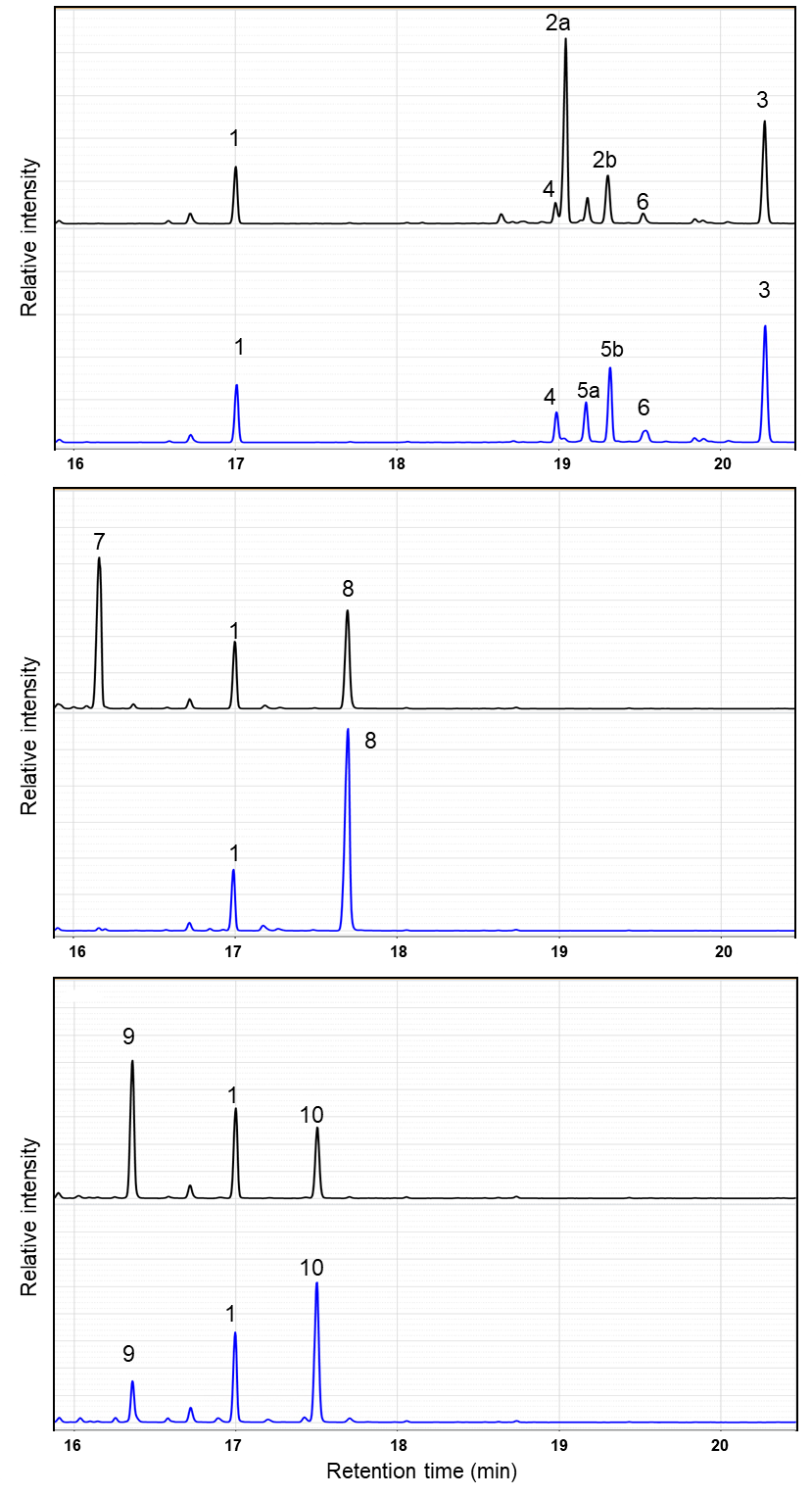


**A**

**B**

**C**

**Fig. S4**. GC-MS analysis of extracellular metabolite composition of KT2440 culture in (**A**) D-galactose, (**B**) L-arabinose, and (**C**) D-ribose approximately 200 hours after inoculation (black line) and at the end (blue line) of the BES experiments. Peak **1**, adonitol-5TMS (internal standard); **2a,b**, galactose-1MOX-5TMS; **3**, galactonic acid-6TMS; **4**, galactono-1,4-lactone-4TMS; **5a**,**b**, 2-keto galactonic acid-1MOX-5TMS; **6**, 2-keto galactonic acid-5TMS; **7**, arabinose-1MOX-4TMS; **8**, arabinonic acid-5TMS; **9**, ribose-1MOX-4TMS; **10**, ribonic acid-5TMS.

**
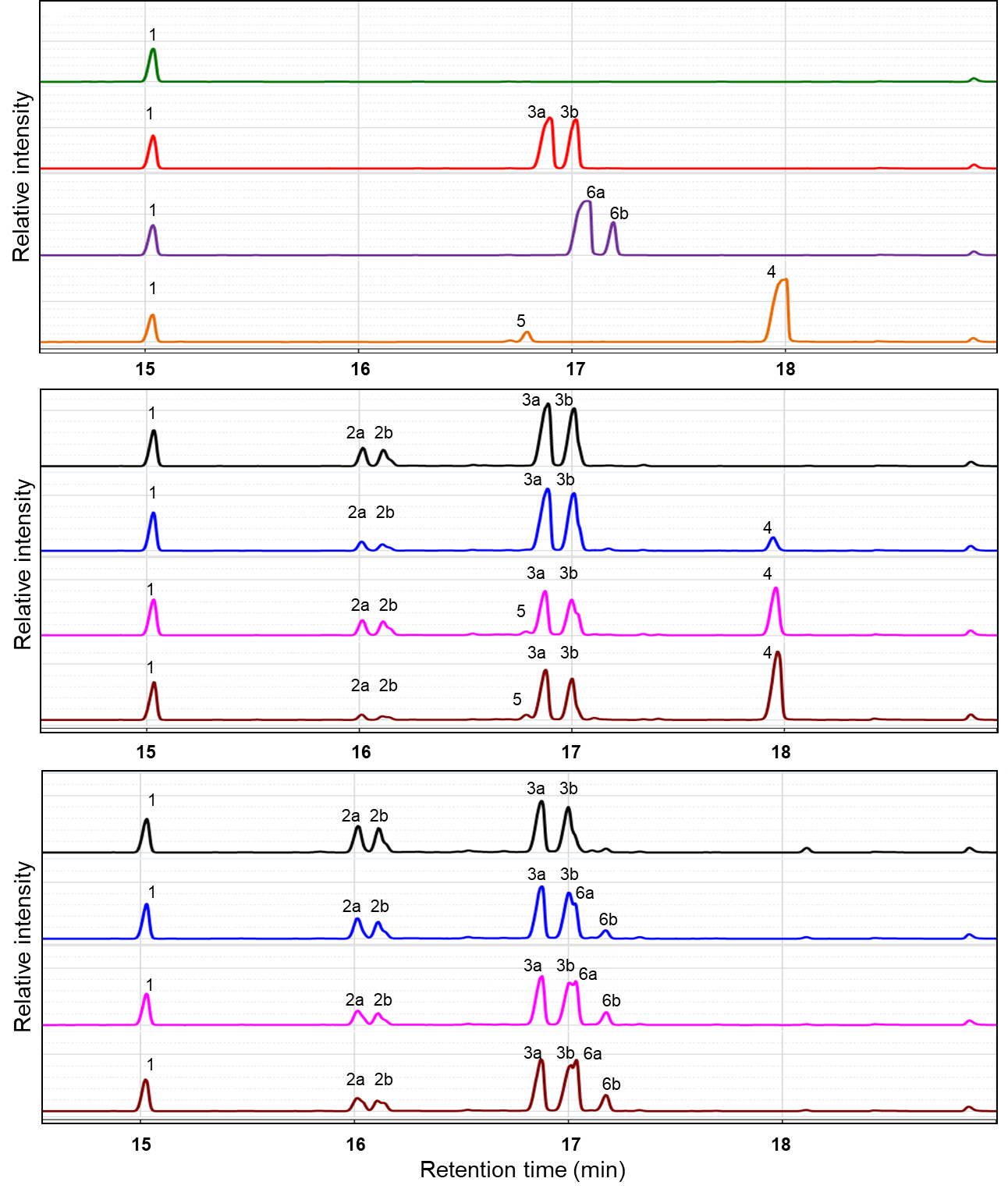
**

**A**

**B**

**C**

**Fig. S5.** GC-MS standard compound identification (**A**, green, adonitol; red, D-fructose; purple, D-mannose; orange, D-mannonate) and analysis of extracellular metabolite composition of (**B**) KT2440’s and (**C**) KT2440Δ*gcd*’s BES cultures at t=2 h (black line), t=146 h (blue line), t = 340 h (magenta line) and t = 480 h (brown line). Peak **1**, adonitol-5TMS (internal standard); **2a**,**b**, fructofuranose-5TMS; **3a**,**b**, fructose-1MOX-5TMS; **4**, mannonic acid-6TMS; **5**, mannono-1,4-lactone-4TMS; **6a**,**b**, mannose-1MOX-5TMS.


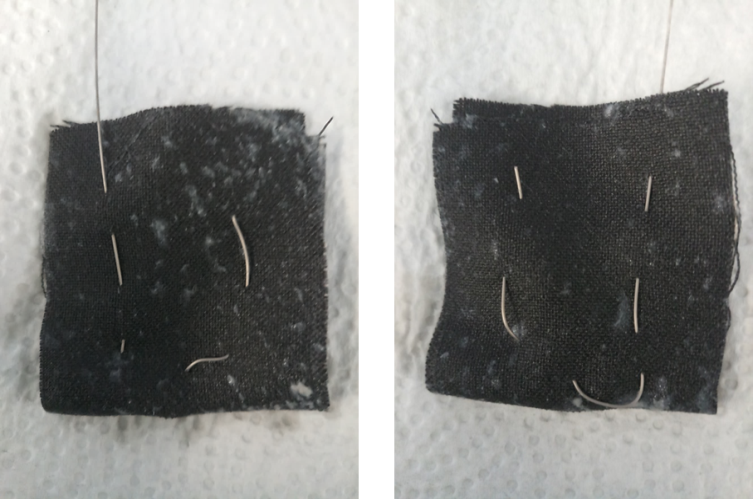


**A**

**B**

**Fig. S6.** Adsorption of *P. putida* (**A**) KT2440’s and (**B**) KT2440Δ*gcd*’s biomasses on anode’s surface.

**
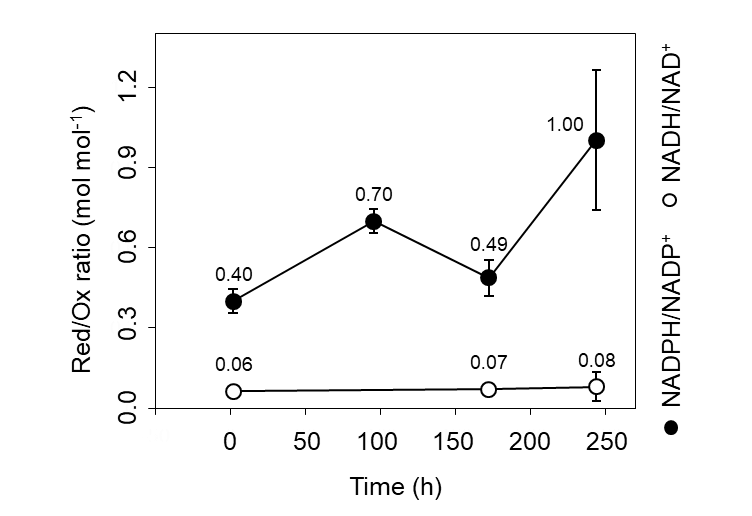
**

**Fig. S7.** NADPH/NADP^+^ and NADH/NAD^+^ ratios in KT2440 cell extracts over the course of their BES cultivation. Data are average of three biological replicates; error bars represent the standard deviation of sample (n=3).


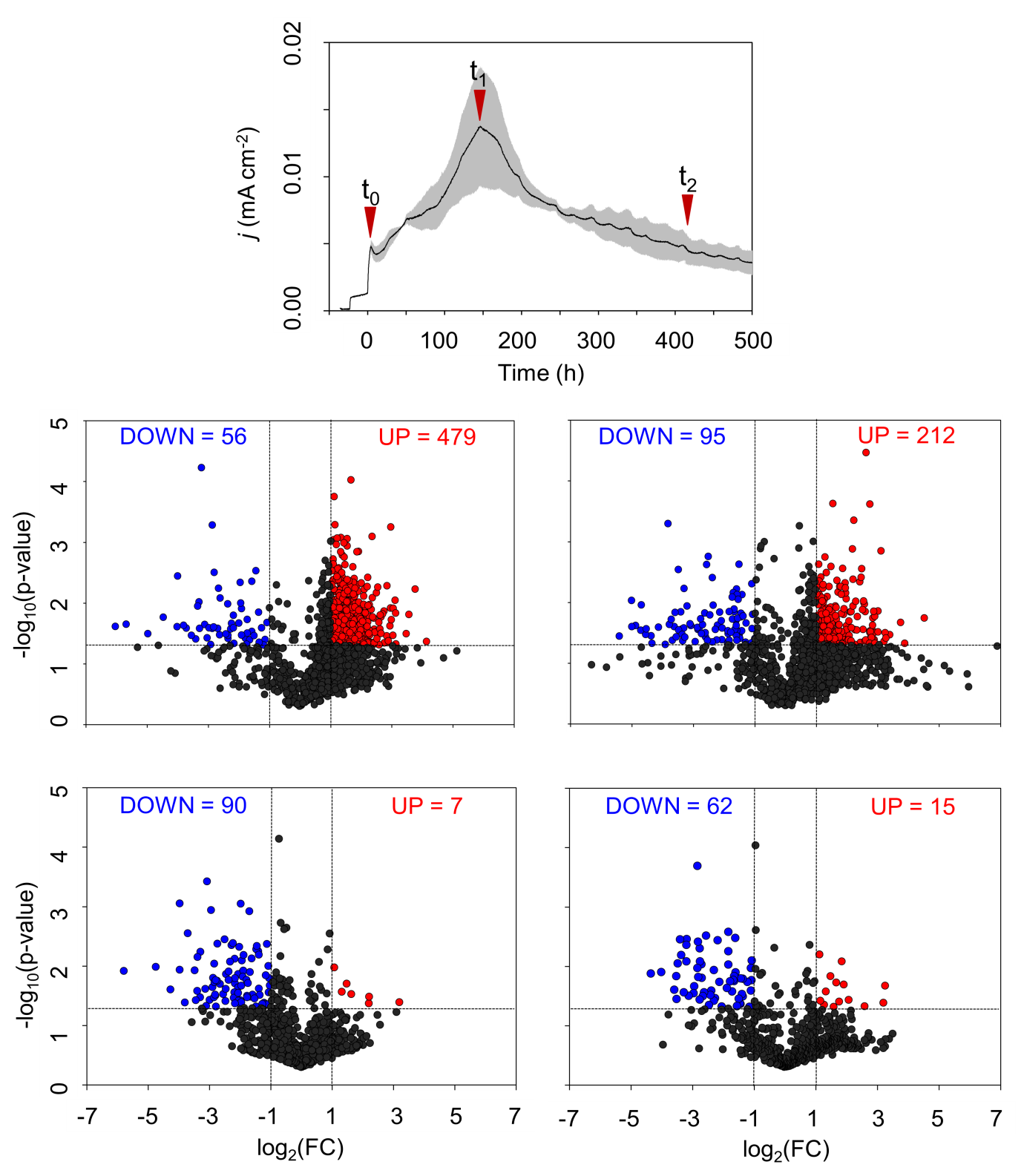


**A**

**B**

**D**

**C**

**E**

**Fig. S8.** (**A**) Current density of KT2440 cultures (n=3) observed during BES cultivation with 2 g L^-1^ fructose and three sampling points for proteomics; KT2440 cultures (n=2) with open-circuit setting were simultaneously sampled at the same time points, using absorbance of ferricyanide as indicator of substrate oxidation. Data are averages of biological replicates; gray area and error bars represent the standard deviations of sample. Volcano plots of KT2440’s proteome during BES cultivation with fructose at t_1_ versus t_0_ (**B**) and t_2_ versus t_0_ (**C**), and volcano plots of KT2440’s proteome during open-circuit cultivation with fructose at t_1_ versus t_0_ (**D**) and t_2_ versus t_0_ (**E**).

**Table S1.** Relative protein abundance of *P. putida* KT2440 during BES (n=3) and open-circuit (n=2) cultivation in fructose

|  | **Protein** | **Locus tag** | **BES^†^** | | | **OC^‡^** | | |
| --- | --- | --- | --- | --- | --- | --- | --- | --- |
|  |  |  | **t_0_** | **t_1_** | **t_2_** | **t_0_** | **t_1_** | **t_2_** |
| ***Fructose***  ***uptake*** | Fructose PTS permease | FruA | 1.271 ± 0.285 | 2.161 ± 0.521 | 1.514 ± 0.500 | 2.378 ± 0.720 | 2.022 ± 0.374 | 2.491 ± 0.205 |
|  | Phosphocarrier protein HPr | FruB | 1.974 ± 0.491 | 4.281 ± 1.224 | 6.416 ± 4.003 | 7.533 ± 1.426 | 7.790 ± 2.328 | 4.781 ± 1.277 |
|  | 1-phosphofructokinase | FruK | 1.973 ± 0.768 | 3.671 ± 1.664 | 2.400 ± 1.304 | 3.288 ± 0.144 | 1.794 ± 2.188 | 0.124 |
| ***Fructose/***  ***mannose***  ***interconversion*** | Aldose-ketose isomerase | YihS | 0.045 ± 0.020 | 0.082 ± 0.035 | 0.033 ± 0.034 | 0.294 | 0.141 | ND |
|  | Mannose-6-phosphate isomerase/mannose-1-phosphate guanylyltransferase | AlgA | ND | ND | ND | ND | ND | 0.288 |
|  |  | PP_1776 | 0.070 ± 0.007 | 0.135 ± 0.059 | 0.593 ± 0.454 | ND | 0.595 ± 0.497 | 0.484 ± 0.371 |
|  |  | PP_4860 | ND | ND | ND | ND | ND | ND |
| ***Glucose/***  ***mannose***  ***transporter*** | Periplasmic binding protein | GtsA | 0.847 ± 0.335 | 0.213 ± 0.142 | 0.048 ± 0.034 | 1.668 ± 0.326 | 0.539 ± 0.001 | 0.503 ± 0.015 |
|  | Permease | GtsBC | 0.002 | ND | ND | ND | ND | ND |
|  | ATP-binding subunit | GtsD | 0.034 ± 0.004 | 0.165 ± 0.073 | 0.087 ± 0.014 | 0.407 ± 0.169 | 0.417 ± 0.458 | 0.316 ± 0.198 |
| ***Peripheral oxidation*** | Glucose dehydrogenase | Gcd | 0.061 ± 0.025 | 0.117 ± 0.020 | 0.083 ± 0.010 | 1.380 ± 0.094 | 1.956 ± 0.597 | 2.272 ± 0.580 |
|  | Gluconate transporter | GntT | ND | ND | 0.010 | ND | ND | ND |
|  |  | PP_0625 | ND | ND | ND | ND | ND | ND |
|  | Gluconate kinase | GnuK | 0.024 ± 0.015 | 0.039 ± 0.026 | 0.054 ± 0.005 | 0.229 | 0.248 ± 0.097 | ND |
|  | Gluconate dehydrogenase complex (Gad) | PP_3382 | ND | ND | ND | ND | ND | ND |
|  |  | PP_3383 | ND | ND | ND | ND | ND | ND |
|  |  | PP_3384 | ND | ND | ND | ND | ND | ND |
|  | 2KGA transporter | KguT | ND | ND | ND | ND | ND | ND |
|  | 2KGA kinase | KguK | ND | 0.0002 | ND | ND | ND | ND |
|  | 2KGA 6-phosphate reductase | KguD | ND | ND | ND | ND | ND | ND |
| ***Embden-Meyerhof-Parnas*** | Hexokinase | Glk | 0.196 ± 0.039 | 0.214 ± 0.070 | 0.354 ± 0.152 | 0.578 ± 0.063 | 0.229 ± 0.014 | 0.303 ± 0.077 |
|  | G6P isomerase | Pgi-1 | ND | 0.258 | 0.329 ± 0.098 | 0.445 ± 0.102 | 1.146 ± 0.308 | 1.668 ± 0.212 |
|  |  | Pgi-2 | 0.446 ± 0.072 | 1.025 ± 0.414 | 0.846 ± 0.194 | 0.462 ± 0.233 | 0.324 ± 0.107 | 0.430 |
|  | F16Pase | Fbp | 0.015 ± 0.013 | 0.020 ± 0.005 | 0.147 ± 0.147 | ND | ND | 0.899 |
|  | F16P aldolase | Fba | 1.175 ± 0.569 | 1.375 ± 0.617 | 0.393 ± 0.150 | 3.128 ± 1.076 | 1.431 ± 0.046 | 1.000 ± 0.938 |
|  | Triose phosphate isomerase | TpiA | 0.372 ± 0.157 | 0.045 | 0.016 ± 0.014 | 0.451 ± 0.101 | 0.250 ± 0.175 | ND |
| ***Pentose phosphate*** | 6PG dehydrogenase | GntZ | 0.210 ± 0.073 | 0.237 ± 0.081 | 0.079 ± 0.048 | 0.415 ± 0.030 | 0.199 ± 0.057 | 0.450 |
|  | RL5P 3-epimerase | Rpe | ND | ND | ND | ND | 0.339 | ND |
|  | R5P isomerase A | RpiA | 0.025 ± 0.006 | 0.014 | 0.027 ± 0.015 | 0.561 ± 0.604 | ND | ND |
|  | Transketolase | TktA | 1.632 ± 0.650 | 3.513 ± 0.781 | 1.795 ± 0.536 | 3.578 ± 0.286 | 2.318 ± 0.567 | 0.683 ± 0.616 |
|  | Transaldolase | Tal | 0.360 ± 0.151 | 0.101 ± 0.067 | 0.125 ± 0.063 | 0.952 ± 0.049 | 0.286 ± 0.237 | 0.116 |
| ***Entner-Doudoroff*** | G6P 1-dehydrogenase | Zwf1 | 0.206 ± 0.061 | 0.415 ± 0.139 | 0.255 ± 0.141 | 0.594 ± 0.257 | 0.457 ± 0.267 | 0.128 |
|  | RL5P 3-epimerase | Zwf2 | 0.018 ± 0.009 | 0.059 ± 0.029 | 0.017 ± 0.005 | ND | ND | ND |
|  | R5P isomerase A | Zwf3 | ND | ND | 0.005 ± 0.004 | 0.970 | ND | ND |
|  | 6-phosphogluconolactonase | Pgl | 0.843 ± 0.308 | 0.179 ± 0.061 | 0.100 ± 0.016 | 2.204 ± 0.336 | 0.425 ± 0.238 | 0.619 ± 0.355 |
|  | 6PG dehydratase | Edd | 0.689 ± 0.053 | 1.360 ± 0.560 | 1.392 ± 0.595 | 1.289 ± 0.007 | 0.660 ± 0.137 | 0.489 |
|  | KDPG aldolase | Eda | 1.098 ± 0.465 | 0.413 ± 0.193 | 0.308 ± 0.055 | 3.199 ± 0.107 | 1.568 ± 0.557 | 0.310 ± 0.024 |
| ***Substrate-level phosphorylation*** | GA3P dehydrogenases | GapA | 1.772 ± 0.601 | 1.964 ± 0.361 | 2.278 ± 1.658 | 6.346 ± 0.882 | 3.901 ± 0.645 | 0.695 ± 0.794 |
|  |  | GapB | 1.288± 0.350 | 2.725 ± 0.455 | 2.743 ± 0.881 | 5.647 ± 0.145 | 6.900 ± 2.437 | 9.377 ± 4.879 |
|  |  | PP_0665 | ND | ND | ND | ND | ND | ND |
|  |  | PP_3443 | 2.169 ± 0.538 | 4.930 ± 1.347 | 3.064 ± 2.134 | 4.278 ± 1.006 | 4.118 ± 0.754 | 2.415 ± 0.989 |
|  | Phosphoglycerate kinase | Pgk | 0.514 ± 0.139 | 0.052 ± 0.026 | 0.101 ± 0.058 | 1.316 ± 0.467 | 0.304 ± 0.036 | 0.155 ± 0.059 |
|  | Phosphoglycerate mutase | Pgm | 0.718 ± 0.333 | 0.278 ± 0.038 | 0.552 ± 0.340 | 1.390 ± 0.034 | 0.353 ± 0.038 | 0.622 |
|  |  | PP_2243 | ND | ND | ND | ND | ND | ND |
|  |  | PP_3923 | 0.001 | 0.011 | 0.007 ± 0.001 | ND | ND | ND |
|  |  | PP_4450 | ND | ND | ND | ND | ND | ND |
|  | Enolase | Eno | 2.375 ± 0.682 | 1.272 ± 1.109 | 1.417 ± 1.975 | 5.584 ± 0.350 | 1.953 ± 0.685 | 0.273 ± 0.036 |
|  | Pyruvate kinase | PykA | 1.146 ± 0.298 | 2.766 ± 0.759 | 1.777 ± 1.090 | 2.254 ± 0.384 | 1.316 ± 0.224 | 0.523 ± 0.184 |
|  |  | Pyk | ND | ND | ND | ND | ND | ND |
| ***Link reaction*** | Pyruvate dehydrogenase complex | AceE | 2.767 ± 1.113 | 5.611 ± 0.158 | 7.467 ± 3.525 | 10.71 ± 1.766 | 10.72 ± 3.061 | 19.58 ± 13.87 |
|  |  | AceF | 4.161 ± 1.418 | 8.616 ± 2.683 | 6.020 ± 1.998 | 1.658 ± 0.341 | 15.18 ± 2.786 | 15.76 ± 1.668 |
|  |  | AcoA | ND | ND | ND | ND | ND | ND |
|  |  | BkdB | 0.151 ± 0.013 | 0.482 ± 0.230 | 0.256 ± 0.175 | 0.441 ± 0.419 | 0.570 ± 0.529 | 0.837 ± 0.763 |
|  |  | Lpd | 0.017 ± 0.005 | 0.017 ± 0.008 | 0.084 ± 0.042 | 0.710 ± 0.234 | 0.204 ± 0.078 | 0.153 |
|  |  | LpdV | 0.196 ± 0.098 | 0.128 ± 0.063 | 0.276 ± 0.290 | 0.170 ± 0.020 | 0.140 ± 0.003 | ND |

**Table S1.** (continued)

|  | **Protein** | **Locus tag** | **BES^†^** | | | **OC^‡^** | | |
| --- | --- | --- | --- | --- | --- | --- | --- | --- |
|  |  |  | **t_0_** | **t_1_** | **t_2_** | **t_0_** | **t_1_** | **t_2_** |
| ***Tricarboxylic acid cycle*** | Citrate synthase | GltA | 0.568 ± 0.115 | 1.104 ± 0.155 | 0.908 ± 0.689 | 1.159 ± 0.000 | 1.459 ± 0.053 | 0.578 ± 0.219 |
|  | Aconitate hydratase | AcnA-I | 0.221 ± 0.084 | 0.310 ± 0.081 | 0.508 ± 0.403 | 0.306 ± 0.025 | 0.157 ± 0.031 | ND |
|  |  | AcnA-II | 0.120 ± 0.075 | 0.238 ± 0.095 | 0.355 ± 0.294 | 0.158 ± 0.045 | 0.165 ± 0.017 | 0.293 ± 0.063 |
|  |  | AcnB | 2.904 ± 0.730 | 5.351 ± 2.130 | 4.849 ± 1.436 | 9.117 ± 0.453 | 5.870 ± 0.060 | 4.071 ± 1.663 |
|  | Isocitrate dehydrogenase | Icd | 2.835 ± 1.187 | 1.932 ± 0.874 | 3.046 ± 1.362 | 4.608 ± 0.309 | 2.619 ± 0.177 | 3.285 ± 0.668 |
|  |  | Idh | 0.319 ± 0.133 | 0.193 ± 0.158 | 0.088 ± 0.036 | 1.241 ± 0.061 | 0.256 ± 0.041 | 0.025 ± 0.145 |
|  | 2-ketoglutarate dehydrogenase complex | SucA | 2.523 ± 0.459 | 6.312 ± 1.187 | 5.071 ± 2.115 | 4.964 ± 0.614 | 6.681 ± 1.012 | 3.726 ± 3.580 |
|  |  | SucB | 4.567 ± 1.620 | 11.85 ± 3.159 | 6.634 ± 1.411 | 5.001 ± 5.421 | 9.157 ± 3.632 | 5.477 ± 6.057 |
|  |  | PP_2652 | ND | ND | ND | ND | ND | ND |
|  |  | PP_3662 | 0.037 ± 0.007 | 0.145 ± 0.102 | 0.178 ± 0.084 | 0.143 ± 0.029 | 0.467 ± 0.311 | 0.281 ± 0.120 |
|  |  | LpdG | 2.774 ± 0.573 | 6.199 ± 2.091 | 4.295 ± 2.484 | 6.963 ± 0.876 | 4.267 ± 3.863 | 3.922 ± 0.089 |
|  | Succinyl coenzyme A synthetase | SucD | 3.036 ± 0.827 | 7.341 ± 1.504 | 5.225 ± 1.163 | 6.257 ± 6.908 | 6.128 ± 0.263 | 0.909 ± 0.301 |
|  |  | SucC | 7.804 ± 1.505 | 17.16 ± 3.975 | 12.42 ± 5.075 | 18.43 ± 1.463 | 12.50 ± 1.400 | 4.045 ± 1.231 |
|  | Succinate dehydrogenase complex | SdhB | 0.325 ± 0.188 | 0.842 ± 0.266 | 1.282 ± 0.373 | 1.396 ± 0.098 | 2.085 ± 1.152 | 0.701 ± 0.584 |
|  |  | SdhA | 1.323 ± 0.201 | 2.201 ± 0.350 | 2.157 ± 0.737 | 4.121 ± 0.350 | 4.454 ± 1.288 | 4.416 ± 1.325 |
|  |  | SdhCD | ND | ND | 0.079 ± 0.060 | 1.329 ± 0.308 | 1.436 ± 0.675 | 1.937 |
|  | Fumarate hydratase | FumC-I | 0.361 ± 0.207 | 0.784 ± 0.438 | 0.534 ± 0.323 | 1.380 ± 0.147 | 1.527 ± 0.411 | 0.430 ± 0.376 |
|  |  | FumC-II | 0.505 ± 0.162 | 1.028 ± 0.308 | 0.310 ± 0.179 | 1.004 ± 0.379 | 1.098 ± 0.510 | 0.708 ± 0.094 |
|  |  | PP_0897 | 0.690 ± 0.218 | 1.567 ± 0.539 | 1.028 ± 0.186 | 1.193 ± 0.430 | 0.970 ± 0.350 | 1.391 ± 0.081 |
|  | Malate dehydrogenase | Mdh | 0.049 ± 0.014 | 0.066 ± 0.029 | ND | 0.399 ± 0.318 | 0.139 | ND |
|  |  | Mqo-1 | 0.043 ± 0.024 | 0.141 ± 0.039 | 0.003 | 0.262 ± 0.152 | 0.637 | ND |
|  |  | Mqo-2 | ND | ND | ND | ND | ND | ND |
|  |  | Mqo-3 | 0.176 ± 0.069 | 0.200 | 0.011 ± 0.011 | 0.353 | ND | ND |
| ***Glyoxyltate***  ***shunt*** | Isocitrate lyase | AceA | 0.216 ± 0.028 | 0.587 ± 0.170 | 0.715 ± 0.522 | 0.571 ± 0.111 | 0.509 ± 0.180 | 1.001 ± 1.237 |
|  | Malate synthase | GlcB | 0.155 ± 0.039 | 0.019 ± 0.019 | 0.055 ± 0.061 | 0.407 ± 0.170 | 0.136 | ND |
| ***Anaplerosis***  ***(Pyruvate shunt)*** | Malic enzyme B | MaeB | 2.075 ± 0.677 | 1.337 ± 1.293 | 0.307 ± 0.069 | 6.707 ± 0.658 | 2.908 ± 0.617 | 0.984 ± 0.786 |
|  | PEP carboxylase | Ppc | 0.166 ± 0.044 | 0.344 ± 0.141 | 0.197 ± 0.053 | 0.546 ± 0.538 | 0.254 ± 0.098 | 0.473 ± 0.273 |
|  | Oxaloacetate decarboxylase | PP_1389 | 0.011 ± 0.013 | 0.019 ± 0.006 | 0.001 | 0.446 ± 0.358 | 0.250 ± 0.129 | ND |
|  | Pyruvate carboxylase | PycA | 0.362 ± 0.143 | 0.826 ± 0.169 | 0.963 ± 0.510 | 0.968 ± 0.363 | 1.054 ± 0.431 | 0.351 ± 0.247 |
|  |  | PycB | 1.341 ± 0.354 | 2.869 ± 0.820 | 1.714 ± 0.529 | 1.223 ± 0.433 | 1.679 ± 0.771 | 0.663 ± 0.034 |
| ***Anaplerosis***  ***(Amino acid catabolism)*** | Glutamate synthase complex | GltB | 0.702 ± 0.041 | 1.923 ± 0.548 | 2.087 ± 1.096 | 1.792 ± 0.074 | 2.806 ± 0.040 | 2.378 ± 1.357 |
|  |  | GltD | 0.203 ± 0.018 | 0.698 ± 0.615 | 0.481 ± 0.155 | 0.214 ± 0.056 | 0.343 ± 0.171 | 0.575 |
|  |  | PP_1060 | ND | 0.017 | 0.018 ± 0.002 | ND | 0.452 | ND |
|  | Glutamate dehydrogenase | GdhA | 0.001 | 0.014 ± 0.011 | 0.009 ± 0.003 | ND | ND | 0.263 |
|  |  | GdhB | 1.531 ± 0.325 | 3.826 ± 1.136 | 4.667 ± 2.627 | 4.191 ± 0.047 | 6.179 ± 2.525 | 12.81 ± 8.973 |
|  | Aspartate ammonia-lyase | AspA | 0.024 ±0.022 | 0.114 ± 0.035 | 0.059 ± 0.034 | ND | 0.889 | ND |
|  | Aspartate aminotranferase | AspC | 0.001 | 0.013 ± 0.006 | 0.007 ± 0.001 | ND | ND | ND |
| The numbers in each box indicate “mean ± standard deviation of sample”; values without standard deviation are of protein detected in only one replicate. t_0_, at inoculation; t_1_, at peak current; t_2_, 414 h after inoculation. ^†^ Normalized to abundance of GapDH Mrsa252 and calculated from 3 biological $\times$ 3 technical replicates; ^‡^ Normalized to abundance of GapDH Mrsa252 and calculated from 2 biological $\times$ 3 technical replicates; ND, not determined. 2KGA, 2-ketogluconate; G6P, glucose 6-phosphate; F16P, fructose 1,6-bisphosphate; 6PG, 6-phosphogluconate; KDPG, 2-keto-3-deoxy-6-phosphogluconate; RL5P, ribulose 5-phosphate; R5P, ribose 5-phosphate; GA3P, glyceraldehyde 3-phosphate; PEP, phosphoenolpyruvate. | | | | | | | | |

**Table S2.** Fold change and significance of central carbon metabolism’s protein expression changes in *P. putida* KT2440 during BES cultivation in fructose (n=3)

|  | **Protein** | **Locus tag** | **t_1_/t_0_** | | **t_2_/t_0_** | |
| --- | --- | --- | --- | --- | --- | --- |
|  |  |  | **Fold change** | **p-value^‡^** | **Fold change** | **p-value^‡^** |
| ***Fructose***  ***uptake*** | Fructose PTS permease | FruA | 1.70 | 0.0143 | 1.19 | 0.0952 |
|  | Phosphocarrier protein HPr | FruB | 2.17 | 0.0203 | 3.25 | 0.0818 |
|  | 1-phosphofructokinase | FruK | 1.86 | 0.1614 | 1.22 | 0.3158 |
| ***Fructose/***  ***mannose***  ***interconversion*** | Aldose-ketose isomerase | YihS | 1.82 | 0.1389 | 0.73 | 0.4910 |
|  | Mannose-6-phosphate isomerase/mannose-1-phosphate guanylyltransferase | AlgA | ND | ND | ND | ND |
|  |  | PP_1776 | 1.92 | 0.1070 | 8.42 | 0.0922 |
|  |  | PP_4860 | ND | ND | ND | ND |
| ***Glucose/***  ***mannose***  ***transporter*** | Periplasmic binding protein | GtsA | 0.25 | 0.0214 | 0.06 | 0.0256 |
|  | Permease | GtsBC | ND | ND | ND | ND |
|  | ATP-binding subunit | GtsD | 4.86 | 0.0440 | 2.56 | 0.0081 |
| ***Peripheral oxidation*** | Glucose dehydrogenase | Gcd | 2.52 | 0.0280 | 2.11 | 0.0109 |
|  | Gluconate transporter | GntT | ND | ND | ND | ND |
|  |  | PP_0625 | ND | ND | ND | ND |
|  | Gluconate kinase | GnuK | 1.60 | 0.4084 | 2.23 | 0.1191 |
|  | Gluconate dehydrogenase complex (Gad) | PP_3382 | ND | ND | ND | ND |
|  |  | PP_3383 | ND | ND | ND | ND |
|  |  | PP_3384 | ND | ND | ND | ND |
|  | 2KGA transporter | KguT | ND | ND | ND | ND |
|  | 2KGA kinase | KguK | ND | ND | ND | ND |
|  | 2KGA 6-phosphate reductase | KguD | ND | ND | ND | ND |
| ***Embden-Meyerhof-Parnas*** | Hexokinase | Glk | 1.10 | 0.2350 | 1.81 | 0.0684 |
|  | G6P isomerase | Pgi-1 | 1.27 | NS | ND | ND |
|  |  | Pgi-2 | 2.30 | 0.0500 | 1.90 | 0.0170 |
|  | F16Pase | Fbp | 1.33 | NS | 9.63 | 0.2410 |
|  | F16P aldolase | Fba | 1.15 | 0.3525 | 0.33 | 0.0424 |
|  | Triose phosphate isomerase | TpiA | 0.12 | NS | 0.04 | 0.0274 |
| ***Pentose phosphate*** | 6PG dehydrogenase | GntZ | 1.13 | 0.3324 | 0.37 | 0.0159 |
|  | RL5P 3-epimerase | Rpe | ND | ND | ND | ND |
|  | R5P isomerase A | RpiA | 0.57 | NS | 1.09 | 0.2576 |
|  | Transketolase | TktA | 2.15 | 0.0009 | 1.10 | 0.1482 |
|  | Transaldolase | Tal | 0.33 | 0.0611 | 0.41 | 0.0494 |
| ***Entner-Doudoroff*** | G6P 1-dehydrogenase | Zwf1 | 2.01 | 0.0391 | 1.09 | 0.3950 |
|  | RL5P 3-epimerase | Zwf2 | 3.21 | 0.0994 | 0.94 | 0.4448 |
|  | R5P isomerase A | Zwf3 | ND | ND | ND | ND |
|  | 6-phosphogluconolactonase | Pgl | 0.21 | 0.0217 | 0.12 | 0.0241 |
|  | 6PG dehydratase | Edd | 1.97 | 0.0968 | 2.02 | 0.0928 |
|  | KDPG aldolase | Eda | 0.38 | 0.0634 | 0.28 | 0.0421 |
| ***Substrate-level phosphorylation*** | GA3P dehydrogenases | GapA | 1.11 | 0.2657 | 1.29 | 0.2718 |
|  |  | GapB | 2.11 | 0.0019 | 2.13 | 0.0243 |
|  |  | PP_0665 | ND | ND | ND | ND |
|  |  | PP_3443 | 2.27 | 0.0170 | 1.41 | 0.2301 |
|  | Phosphoglycerate kinase | Pgk | 0.10 | 0.0096 | 0.20 | 0.0111 |
|  | Phosphoglycerate mutase | Pgm | 0.39 | 0.0653 | 0.77 | 0.1389 |
|  |  | PP_2243 | ND | ND | ND | ND |
|  |  | PP_3923 | 14.04 | NS | 8.29 | NS |
|  |  | PP_4450 | ND | ND | ND | ND |
|  | Enolase | Eno | 0.54 | 0.1107 | 0.60 | 0.0723 |
|  | Pyruvate kinase | PykA | 2.41 | 0.0132 | 1.55 | 0.1629 |
|  |  | Pyk | ND | ND | ND | ND |
| ***Link reaction*** | Pyruvate dehydrogenase complex | AceE | 2.03 | 0.0302 | 2.70 | 0.0483 |
|  |  | AceF | 2.07 | 0.0166 | 1.45 | 0.0152 |
|  |  | AcoA | ND | ND | ND | ND |
|  |  | BkdB | 3.20 | 0.0626 | 1.70 | 0.1909 |
|  |  | Lpd | 0.96 | 0.2593 | 4.79 | 0.1233 |
|  |  | LpdV | 0.65 | 0.1840 | 1.41 | 0.3371 |

**Table S2.** (continued)

|  | **Protein** | **Locus tag** | **t_1_/t_0_** | | **t_2_/t_0_** | |
| --- | --- | --- | --- | --- | --- | --- |
|  |  |  | **Fold change** | **p-value^‡^** | **Fold change** | **p-value^‡^** |
| ***Tricarboxylic acid cycle*** | Citrate synthase | GltA | 1.94 | 0.0152 | 1.60 | 0.2098 |
|  | Aconitate hydratase | AcnA-I | 1.40 | 0.0678 | 2.29 | 0.1317 |
|  |  | AcnA-II | 1.97 | 0.0252 | 2.95 | 0.1061 |
|  |  | AcnB | 1.84 | 0.0506 | 1.67 | 0.0416 |
|  | Isocitrate dehydrogenase | Icd | 0.68 | 0.0244 | 1.07 | 0.2689 |
|  |  | Idh | 0.61 | 0.0222 | 0.27 | 0.0329 |
|  | 2-ketoglutarate dehydrogenase complex | SucA | 2.50 | 0.0061 | 2.01 | 0.0584 |
|  |  | SucB | 2.60 | 0.0091 | 1.45 | 0.0274 |
|  |  | PP_2652 | ND | ND | ND | ND |
|  |  | PP_3662 | 3.88 | 0.0993 | 4.76 | 0.0492 |
|  |  | LpdG | 2.23 | 0.0323 | 1.55 | 0.1575 |
|  | Succinyl coenzyme A synthetase | SucD | 2.42 | 0.0094 | 1.72 | 0.0054 |
|  |  | SucC | 2.20 | 0.0112 | 1.59 | 0.0820 |
|  | Succinate dehydrogenase complex | SdhB | 2.59 | 0.0403 | 3.95 | 0.0087 |
|  |  | SdhA | 1.66 | 0.0131 | 1.63 | 0.0575 |
|  |  | SdhCD | ND | ND | ND | ND |
|  | Fumarate hydratase | FumC-I | 2.17 | 0.0488 | 1.48 | 0.1226 |
|  |  | FumC-II | 2.04 | 0.0126 | 0.61 | 0.0193 |
|  |  | PP_0897 | 2.27 | 0.0209 | 1.49 | 0.0036 |
|  | Malate dehydrogenase | Mdh | 1.35 | 0.2439 | ND | ND |
|  |  | Mqo-1 | 3.30 | 0.0222 | 0.08 | NS |
|  |  | Mqo-2 | ND | ND | ND | ND |
|  |  | Mqo-3 | 1.14 | NS | 0.06 | 0.0773 |
| ***Glyoxyltate***  ***shunt*** | Isocitrate lyase | AceA | 2.72 | 0.0233 | 3.32 | 0.1114 |
|  | Malate synthase | GlcB | 0.12 | 0.0574 | 0.36 | 0.0393 |
| ***Anaplerosis***  ***(Pyruvate shunt)*** | Malic enzyme B | MaeB | 0.64 | 0.2065 | 0.15 | 0.0187 |
|  | PEP carboxylase | Ppc | 2.07 | 0.0441 | 1.18 | 0.1151 |
|  | Oxaloacetate decarboxylase | PP_1389 | 1.63 | 0.2612 | 0.10 | NS |
|  | Pyruvate carboxylase | PycA | 2.28 | 0.0011 | 2.66 | 0.0527 |
|  |  | PycB | 2.14 | 0.0150 | 1.28 | 0.0333 |
| ***Anaplerosis***  ***(Amino acid catabolism)*** | Glutamate synthase complex | GltB | 2.74 | 0.0270 | 2.97 | 0.0755 |
|  |  | GltD | 3.45 | 0.0190 | 2.37 | 0.0373 |
|  |  | PP_1060 | ND | ND | ND | ND |
|  | Glutamate dehydrogenase | GdhA | 10.47 | NS | 6.65 | NS |
|  |  | GdhB | 2.50 | 0.0198 | 3.05 | 0.0713 |
|  | Aspartate ammonia-lyase | AspA | 4.77 | 0.0317 | 2.47 | 0.3142 |
|  | Aspartate aminotranferase | AspC | 12.65 | NS | 7.21 | NS |
| t_0_, at inoculation; t_1_, at peak current; t_2_, 414 h after inoculation. ^‡^ Statistical analysis of protein abundance over the course of the experiment was conducted using one-tailed paired t-test; ND, not determined due to the lack of data; NS, sample size not sufficient for statistical test (protein detected in less than two dependent samples). 2KGA, 2-ketogluconate; G6P, glucose 6-phosphate; F16P, fructose 1,6-bisphosphate; 6PG, 6-phosphogluconate; KDPG, 2-keto-3-deoxy-6-phosphogluconate; RL5P, ribulose 5-phosphate; R5P, ribose 5-phosphate; GA3P, glyceraldehyde 3-phosphate; PEP, phosphoenolpyruvate. | | | | | | |

**Table S3.** Fold change and significance of central carbon metabolism’s protein expression changes in *P. putida* KT2440 during open-circuit cultivation in fructose (n=2)

|  | **Protein** | **Locus tag** | **t_1_/t_0_** | | **t_2_/t_0_** | |
| --- | --- | --- | --- | --- | --- | --- |
|  |  |  | **Fold change** | **p-value^‡^** | **Fold change** | **p-value^‡^** |
| ***Fructose***  ***uptake*** | Fructose PTS permease | FruA | 0.85 | 0.1916 | 1.05 | 0.4040 |
|  | Phosphocarrier protein HPr | FruB | 1.03 | 0.3784 | 0.63 | 0.1932 |
|  | 1-phosphofructokinase | FruK | 0.55 | 0.2447 | 0.02 | NS |
| ***Fructose/***  ***mannose***  ***interconversion*** | Aldose-ketose isomerase | YihS | 0.48 | NS | ND | ND |
|  | Mannose-6-phosphate isomerase/mannose-1-phosphate guanylyltransferase | AlgA | ND | ND | ND | ND |
|  |  | PP_1776 | ND | ND | ND | ND |
|  |  | PP_4860 | ND | ND | ND | ND |
| ***Glucose/***  ***mannose***  ***transporter*** | Periplasmic binding protein | GtsA | 0.32 | 0.0638 | 0.30 | 0.0594 |
|  | Permease | GtsBC | ND | ND | ND | ND |
|  | ATP-binding subunit | GtsD | 1.16 | 0.4542 | 0.78 | 0.0719 |
| ***Peripheral oxidation*** | Glucose dehydrogenase | Gcd | 1.42 | 0.1760 | 1.65 | 0.1561 |
|  | Gluconate transporter | GntT | ND | ND | ND | ND |
|  |  | PP_0625 | ND | ND | ND | ND |
|  | Gluconate kinase | GnuK | 2.16 | NS | ND | ND |
|  | Gluconate dehydrogenase complex (Gad) | PP_3382 | ND | ND | ND | ND |
|  |  | PP_3383 | ND | ND | ND | ND |
|  |  | PP_3384 | ND | ND | ND | ND |
|  | 2KGA transporter | KguT | ND | ND | ND | ND |
|  | 2KGA kinase | KguK | ND | ND | ND | ND |
|  | 2KGA 6-phosphate reductase | KguD | ND | ND | ND | ND |
| ***Embden-Meyerhof-Parnas*** | Hexokinase | Glk | 0.39 | 0.0307 | 0.52 | 0.0111 |
|  | G6P isomerase | Pgi-1 | 2.58 | 0.1248 | 3.75 | 0.0202 |
|  |  | Pgi-2 | 0.70 | 0.3338 | 0.46 | NS |
|  | F16Pase | Fbp | ND | ND | ND | ND |
|  | F16P aldolase | Fba | 0.46 | 0.1290 | 0.32 | 0.0146 |
|  | Triose phosphate isomerase | TpiA | 0.55 | 0.0806 | ND | ND |
| ***Pentose phosphate*** | 6PG dehydrogenase | GntZ | 0.48 | 0.0278 | 0.54 | NS |
|  | RL5P 3-epimerase | Rpe | ND | ND | ND | ND |
|  | R5P isomerase A | RpiA | ND | ND | ND | ND |
|  | Transketolase | TktA | 0.65 | 0.0498 | 0.19 | 0.0256 |
|  | Transaldolase | Tal | 0.30 | 0.0626 | 0.06 | NS |
| ***Entner-Doudoroff*** | G6P 1-dehydrogenase | Zwf1 | 0.77 | 0.0174 | 0.11 | NS |
|  | RL5P 3-epimerase | Zwf2 | ND | ND | ND | ND |
|  | R5P isomerase A | Zwf3 | ND | ND | ND | ND |
|  | 6-phosphogluconolactonase | Pgl | 0.19 | 0.0125 | 0.28 | 0.0026 |
|  | 6PG dehydratase | Edd | 0.51 | 0.0513 | 0.19 | NS |
|  | KDPG aldolase | Eda | 0.49 | 0.0614 | 0.10 | 0.0065 |
| ***Substrate-level phosphorylation*** | GA3P dehydrogenases | GapA | 0.61 | 0.0218 | 0.11 | 0.0035 |
|  |  | GapB | 1.22 | 0.2905 | 1.66 | 0.2328 |
|  |  | PP_0665 | ND | ND | ND | ND |
|  |  | PP_3443 | 0.96 | 0.4593 | 0.56 | 0.2063 |
|  | Phosphoglycerate kinase | Pgk | 0.23 | 0.0931 | 0.12 | 0.0986 |
|  | Phosphoglycerate mutase | Pgm | 0.25 | 0.0009 | 0.22 | NS |
|  |  | PP_2243 | ND | ND | ND | ND |
|  |  | PP_3923 | ND | ND | ND | ND |
|  |  | PP_4450 | ND | ND | ND | ND |
|  | Enolase | Eno | 0.35 | 0.0617 | 0.05 | 0.0133 |
|  | Pyruvate kinase | PykA | 0.58 | 0.0382 | 0.23 | 0.0726 |
|  |  | Pyk | ND | ND | ND | ND |
| ***Link reaction*** | Pyruvate dehydrogenase complex | AceE | 1.00 | 0.4968 | 1.83 | 0.2443 |
|  |  | AceF | 9.16 | 0.0405 | 9.50 | 0.0212 |
|  |  | AcoA | ND | ND | ND | ND |
|  |  | BkdB | 1.29 | 0.1733 | 1.90 | 0.3592 |
|  |  | Lpd | 0.29 | 0.0684 | 0.11 | NS |
|  |  | LpdV | 0.82 | 0.1218 | ND | ND |

**Table S3.** (continued)

|  | **Protein** | **Locus tag** | **t_1_/t_0_** | | **t_2_/t_0_** | |
| --- | --- | --- | --- | --- | --- | --- |
|  |  |  | **Fold change** | **p-value^‡^** | **Fold change** | **p-value^‡^** |
| ***Tricarboxylic acid cycle*** | Citrate synthase | GltA | 1.26 | 0.0395 | 0.50 | 0.0829 |
|  | Aconitate hydratase | AcnA-I | 0.51 | 0.0828 | ND | ND |
|  |  | AcnA-II | 1.04 | 0.4531 | 1.86 | 0.1631 |
|  |  | AcnB | 0.64 | 0.0272 | 0.45 | 0.0918 |
|  | Isocitrate dehydrogenase | Icd | 0.57 | 0.0149 | 0.71 | 0.0603 |
|  |  | Idh | 0.21 | 0.0044 | 0.18 | 0.0452 |
|  | 2-ketoglutarate dehydrogenase complex | SucA | 1.35 | 0.0517 | 0.75 | 0.3742 |
|  |  | SucB | 1.83 | 0.3167 | 1.10 | 0.4818 |
|  |  | PP_2652 | ND | ND | ND | ND |
|  |  | PP_3662 | 3.72 | 0.1757 | 1.97 | 0.2079 |
|  |  | LpdG | 0.61 | 0.2116 | 0.56 | 0.0702 |
|  | Succinyl coenzyme A synthetase | SucD | 0.99 | 0.4952 | 0.15 | 0.2424 |
|  |  | SucC | 0.68 | 0.0024 | 0.22 | 0.0036 |
|  | Succinate dehydrogenase complex | SdhB | 1.49 | 0.2893 | 0.50 | 0.1463 |
|  |  | SdhA | 1.08 | 0.3521 | 1.07 | 0.3715 |
|  |  | SdhCD | 1.08 | 0.3756 | 0.73 | NS |
|  | Fumarate hydratase | FumC-I | 1.11 | 0.2875 | 0.31 | 0.1181 |
|  |  | FumC-II | 1.09 | 0.2472 | 0.71 | 0.2699 |
|  |  | PP_0897 | 0.81 | 0.3779 | 1.17 | 0.3406 |
|  | Malate dehydrogenase | Mdh | 0.17 | NS | ND | ND |
|  |  | Mqo-1 | 1.22 | NS | ND | ND |
|  |  | Mqo-2 | ND | ND | ND | ND |
|  |  | Mqo-3 | ND | ND | ND | ND |
| ***Glyoxyltate***  ***shunt*** | Isocitrate lyase | AceA | 0.89 | 0.2102 | 1.75 | 0.3423 |
|  | Malate synthase | GlcB | 0.17 | NS | ND | ND |
| ***Anaplerosis***  ***(Pyruvate shunt)*** | Malic enzyme B | MaeB | 0.43 | 0.0742 | 0.15 | 0.0050 |
|  | PEP carboxylase | Ppc | 0.46 | 0.3166 | 0.87 | 0.3809 |
|  | Oxaloacetate decarboxylase | PP_1389 | 0.56 | 0.2206 | ND | ND |
|  | Pyruvate carboxylase | PycA | 1.09 | 0.1618 | 0.36 | 0.0421 |
|  |  | PycB | 1.37 | 0.1534 | 0.54 | 0.1697 |
| ***Anaplerosis***  ***(Amino acid catabolism)*** | Glutamate synthase complex | GltB | 1.57 | 0.0253 | 1.33 | 0.3330 |
|  |  | GltD | 1.60 | 0.2845 | 1.35 | NS |
|  |  | PP_1060 | ND | ND | ND | ND |
|  | Glutamate dehydrogenase | GdhA | ND | ND | ND | ND |
|  |  | GdhB | 1.47 | 0.2299 | 3.06 | 0.2011 |
|  | Aspartate ammonia-lyase | AspA | ND | ND | ND | ND |
|  | Aspartate aminotranferase | AspC | ND | ND | ND | ND |
| t_0_, at inoculation; t_1_, at peak current; t_2_, 414 h after inoculation. ^‡^ Statistical analysis of protein abundance over the course of the experiment was conducted using one-tailed paired t-test; ND, not determined due to the lack of data; NS, sample size not sufficient for statistical test (protein detected in less than two replicates). 2KGA, 2-ketogluconate; G6P, glucose 6-phosphate; F16P, fructose 1,6-bisphosphate; 6PG, 6-phosphogluconate; KDPG, 2-keto-3-deoxy-6-phosphogluconate; RL5P, ribulose 5-phosphate; R5P, ribose 5-phosphate; GA3P, glyceraldehyde 3-phosphate; PEP, phosphoenolpyruvate. | | | | | | |

**Table S4.** Differential expression analysis of selected regulatory proteins of the CCM in *P.putida* KT2440 during BES cultivation in fructose

| **Regulator** | **Fold change** | | | **Description** | | | |
| --- | --- | --- | --- | --- | --- | --- | --- |
|  | **t_1_/t_0_** | **t_2_/t_0_** | **Role** | | **Effector** | **Target gene/operon/protein** | **Reference** |
| Cra | 3.08^*^ | 2.10^*^ | R | | F1P | *fruBKA, PP_3443* | Chavarría, et al. (2016) |
| PtxS | ^ND^ | ^ND^ | R | | 2KGA | *ptxS, kguEKTD,  PP_3382-3384* (*gadCBA*) | del Castillo, et al. (2008), Daddaoua, et al. (2010), Udaondo, et al. (2018) |
| HexR | 1.56 | 1.76 | R | | KDPG | *gap-1, edd-glk-gltR-2,  zwf-pgl-eda* | del Castillo, et al. (2008), Kim, et al. (2008), Daddaoua, et al. (2009), Campilongo, et al. (2017), Udaondo, et al. (2018) |
| FnrA | 1.54 ^NS^ | 28.7 ^NS^ | R/A | | Unknown | *gap, edd, hexR, gnuR* | Tribelli, et al. (2019) |
| GltR-II | 2.48^***^ | 2.17^**^ | A | | 2KGA/6PG | *oprB, gtsABCD* | del Castillo, et al. (2008), Nikel, et al. (2014a) |
| AceK | 25.1^NS^ | 24.5^NS^ | I | | Unknown | Isocitrate dehydrogenase (Icd) | Crousilles, et al. (2018) |
| t_0_, at inoculation; t_1_, at peak current; t_2_, 414 h after inoculation. ND, not determined; *, P (p-value) < 0.05; **, P < 0.01; ***, P < 0.001; NS, sample size not sufficient for statistical test (protein detected in less than two replicates); R, transcriptional repressor; A, transcriptional activator; I, protein inactivator (via phosphorylation). F1P, fructose 1-phosphate; 2KGA, 2-ketogluconate; KDPG, 2-keto-3-deoxy-6-phosphogluconate; 6PG, 6-phosphogluconate. | | | | | | | |
